# The H3.3 chaperone Hira complex orchestrates oocyte developmental competence

**DOI:** 10.1242/dev.200044

**Published:** 2022-02-28

**Authors:** Rowena Smith, Andrej Susor, Hao Ming, Janet Tait, Marco Conti, Zongliang Jiang, Chih-Jen Lin

**Affiliations:** 1 MRC Centre for Reproductive Health, University of Edinburgh, Queen's Medical Research Institute, 47 Little France Crescent, Edinburgh EH16 4TJ, UK; 2 Laboratory of Biochemistry and Molecular Biology of Germ Cells, Institute of Animal Physiology and Genetics, CAS, Rumburska 89, 277 21 Libechov, Czech Republic; 3 School of Animal Sciences, AgCenter, Louisiana State University, Baton Rouge, LA 70803, USA; 4 Center for Reproductive Sciences, University of California, San Francisco, CA 94143, USA

**Keywords:** Hira complex, Histone H3.3, Oocyte-to-embryo transition, Zygotic genome activation, Competent oocyte

## Abstract

Successful reproduction requires an oocyte competent to sustain early embryo development. By the end of oogenesis, the oocyte has entered a transcriptionally silenced state, the mechanisms and significance of which remain poorly understood. Histone H3.3, a histone H3 variant, has unique cell cycle-independent functions in chromatin structure and gene expression. Here, we have characterised the H3.3 chaperone Hira/Cabin1/Ubn1 complex, showing that loss of function of any of these subunits causes early embryogenesis failure in mouse. Transcriptome and nascent RNA analyses revealed that transcription is aberrantly silenced in mutant oocytes. Histone marks, including H3K4me3 and H3K9me3, are reduced and chromatin accessibility is impaired in Hira/Cabin1 mutants. Misregulated genes in mutant oocytes include *Zscan4d*, a two-cell specific gene involved in zygote genome activation. Overexpression of *Zscan4* in the oocyte partially recapitulates the phenotypes of Hira mutants and *Zscan4* knockdown in *Cabin1* mutant oocytes partially restored their developmental potential, illustrating that temporal and spatial expression of Zscan4 is fine-tuned at the oocyte-to-embryo transition. Thus, the H3.3 chaperone Hira complex has a maternal effect function in oocyte developmental competence and embryogenesis, through modulating chromatin condensation and transcriptional quiescence.

## Abstract (precis)

**Summary:** The H3.3 chaperone Hira complex has a maternal effect function in oocyte developmental competence and embryogenesis by modulating chromatin condensation and transcriptional quiescence.

## INTRODUCTION

Oocytes are key orchestrators of fertilisation, initiators of zygotic genome activation (ZGA) and pre-implantation development (Conti and Franciosi, 2018). This potential is defined as developmental competence. Oocytes defective in developmental competence are a major cause of infertility, a medical condition that affects one in six couples. One hallmark feature of a competent oocyte is chromatin condensation. During the final stage of oocyte growth, oocytes with less condensed chromatin, termed non-surrounded nucleolus (NSN) oocytes, gradually transition to more-condensed, surrounded nucleolus (SN) oocytes (Zuccotti et al., 1995). Transcriptional silencing occurs around this time (De La Fuente et al., 2004). However, the molecular components involved have not been defined and the primary underlying mechanism remains elusive (Schultz et al., 2018).

Oocytes enter a protracted meiotic arrest in prophase I but, remarkably, retain their competence and genomic integrity for a long time, for years in humans. It would then be logical to hypothesise that replication-independent mechanisms of chromatin regulation may be indispensable to maintaining the quality of oocytes throughout this prolonged meiotic arrest.

Histones are the fundamental blocks of chromatin that regulate both chromatin compactness and gene transcriptional processes. In addition to canonical histones, histone variants provide additional layers of regulatory mechanisms in a replication-independent manner. H3.3 is the primary replication-independent histone variant and has been shown to play prominent roles during crucial stages of reproduction, including fertilisation (Lin et al., 2013; Inoue and Zhang, 2014) and early embryonic development (Lin et al., 2013; Wen et al., 2014). However, potential roles of H3.3 in oocyte transcriptional quiescence remain unexplored.

The key chaperone molecules required for H3.3 incorporation into chromatin form the Hira complex, which comprises Hira, Ubn1 and Cabin1 subunits (Ray-Gallet et al., 2018; Rai et al., 2011). In this study, we comprehensively dissect the roles of the Hira complex (Hira, Cabin1 and Ubn1) in establishment and maintenance of developmental competence in mouse oocytes. We demonstrate that the Hira complex is crucial for oocyte developmental competence through stabilisation of a repressive epigenomic status that maintains transcriptional quiescence in preparation for embryogenesis.

## RESULTS

### The maternal Hira complex is essential for embryo development

To investigate the maternal role of the Hira complex during the oocyte-to-embryo transition (Schultz et al., 2018), we applied a loss-of-function approach where each of the three subunits of the complex are individually inactivated. To do this, we inhibited Ubn1 translation in fully grown, germinal vesicle (GV) oocytes using morpholino microinjection (Lin et al., 2014; Fig. S1A). Conditional knockout mouse lines targeting Hira and Cabin1 were generated by crossing a transgenic Zp3-Cre mouse line (de Vries et al., 2000) with a conditional allele of Hira to deplete exon 6-7 (hereafter referred to as ZH; Fig. S1B) and a conditional allele of Cabin1 to deplete of exon 6 (hereafter referred to as CabZ; Fig. S1C), respectively.

**Fig. 1.**
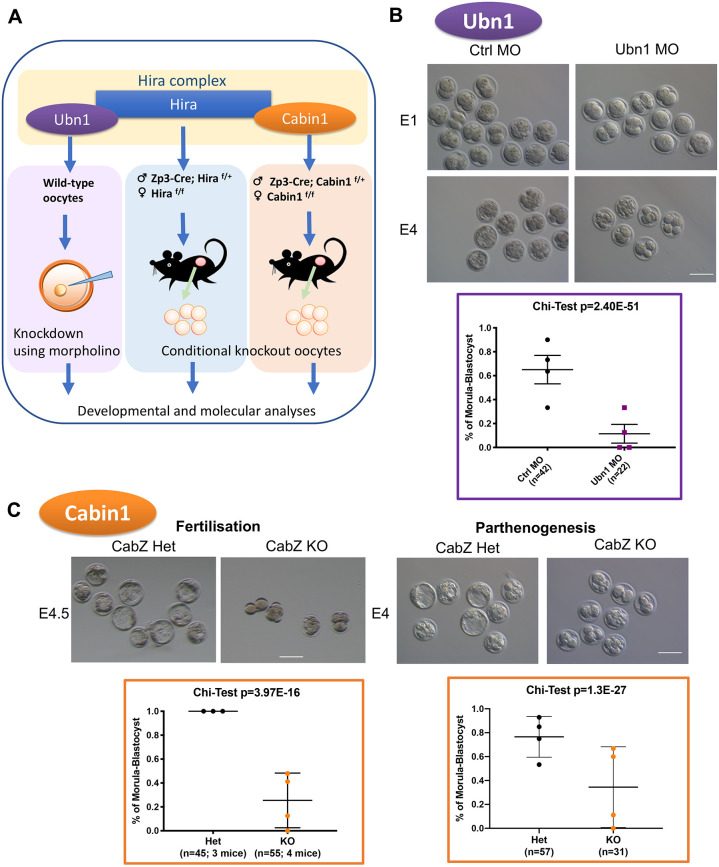
**The maternal Hira complex is essential for pre-implantation development.** (A) Oocyte-specific loss-of-function approaches for each subunit of the Hira complex. Ubn1 was knocked down by microinjection of morpholino antisense oligonucleotides. Hira and Cabin1 knockout mice were generated by crossing a Zp3-Cre line with Hira^flox/flox^ (ZH) and Cabin1^flox/flox^ (CabZ) lines, respectively. (B) Ubn1 is required for pre-implantation development of parthenogenetic embryos. Developmental progression of control (Ctrl) and Ubn1 morpholino (MO) injected embryos at embryonic day 1 (E1) and E4. Representative bright-field images and quantification. Scale bar: 100 µm. Data are mean±s.d.(C) Both fertilised and parthenogenetically activated Cabin1 mutant oocytes arrested during preimplantation development. Morula-to-blastocyst developmental potential of fertilised mutant Cabin1 embryos is impaired. Bright-field images and quantification of development. Data are mean±s.d. Scale bar: 80 µm.

To investigate whether Ubn1 is involved in oocyte developmental competence, we compared the phenotypes of oocytes injected with control morpholino (Ctrl MO) with those injected with Ubn1 morpholino (Ubn1 MO). Microinjected GV oocytes then underwent *in vitro* maturation to the MII stage followed by parthenogenesis and pre-implantation development to the morula-to-blastocyst stage (Fig. S1A). Oocyte depletion of Ubn1 impaired maturation (55%, compared with Ctrl MO group 65%; *P*=1.7E-04; Fig. S1D). Importantly, it also induced a significant decrease in cleavage to two-cell stage (47%, compared with Ctrl MO group 78%; *P*=3.4E-06; Fig. S1E) as well as development to morula-to-blastocyst stage (11%, compared with Ctrl MO group 65%; *P*=2.4E-51; Fig. 1B).

Both ZH and CabZ mutant mice are infertile, showing the similar phenotype as our previously reported Hira mutants (Lin et al., 2014). We have recently reported the impairment of H3.3 incorporation and the abnormal male pronucleus formation phenotypes of Hira, Cabin1 and Ubn1 in a separate study (Smith et al., 2021). Interestingly, we noted that mice had decreased fertility in the heterozygous group (Zp3-Cre; Cabin1 flox/+) compared with the wild-type controls (either Cabin1 flox/flox or flox/+ without Zp3-Cre) after mating with a wild-type male (average litter size of five from the heterozygous females compared with 8.3 from the wild-type females, *P*=0.02). In order to minimise the effect of variable genetic background, for all subsequent experiments we used littermates from heterozygous mating of ZH and Cabz mice.

To examine the competence of oocytes depleted of Cabin1, first we flushed fertilised zygotes from CabZ heterozygous controls (CabZ Het) and mutants (CabZ KO), then monitored their developmental outcomes and compared the rate of development to morula-to-blastocyst stage embryos after *in vitro* culture. CabZ KO embryos had significantly reduced ability to form morula-to-blastocyst stage embryos (25%, compared with CabZ Het group of 100%; *P*=4E-16): the majority arrested at the two- to four-cell stages (Fig. 1C), which suggests a defect in ZGA (Bultman et al., 2006; Kim et al., 2016). Second, we performed parthenogenetic activation on *in vitro* matured MII oocytes from CabZ Het and CabZ KO mice. Parthenogenetic CabZ KO embryos failed to develop to morula-to-blastocyst stage and arrested at the two- to four-cell stage (34%, compared with CabZ Het group of 77%; *P*=1.3E-27; Fig. 1C). These results firmly support the observation that the developmental competence of mutant Cabin1 oocytes is compromised, as assessed both by *in vivo* fertilisation and by parthenogenetic activation, and therefore lacks contribution from the paternal genome.

The above genetic manipulations consistently show that loss of any of the components of the Hira complex produces a phenotype of disruption of early embryo development. This conclusion is further supported by the depletion of the target of the chaperone Hira complex: histone H3.3. Using the same approach used for Ubn1 knockdown, we interfered with H3.3 mRNA translation in wild-type GV oocytes. In agreement with the loss-of-function of the H3.3 chaperone Hira complex (Hira, Cabin1 and Ubn1), H3.3 depletion in oocytes resulted in the developmental arrest of parthenogenetic embryos with a significantly impaired formation of both two-cell stage (74%, compared with Ctrl MO group of 91%; *P*=5.7E-5; Fig. S1F) and later morula-to-blastocyst stage (6%, compared with Ctrl MO group of 87%; *P*=1.5E-124; Fig. S1F) embryos.

### Several ZGA genes are depressed in Hira and Cabin1 KO oocytes

**Fig. 2.**
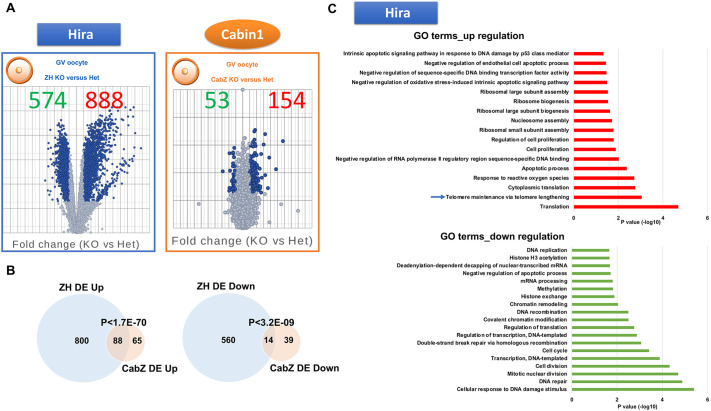
**Loss of the Hira complex impairs global transcription in GV oocytes.** (A) Volcano plots showed differentially expressed genes of Hira and Cabin1 mutant (ZH KO and CabZ KO) oocytes. Left panel: differentially expressed genes of ZH KO compared with ZH Het oocytes. Right panel: differentially expressed genes of CabZ KO compared with CabZ Het oocytes. (B) Venn diagrams reveal the subsets of co-regulated genes dysregulated in ZH KO and CabZ KO oocytes. For comparison, the differential expression cut-off threshold has a false discovery rate (FDR) adjusted *P*<0.05 and fold change >2. Significance of overlap was assessed by hypergeometric test. (C) Top GO terms from up- and downregulated genes of Hira mutant oocytes.

Taken together, the above results demonstrate that loss of function of any one of the subunits of the H3.3-Hira complex in oocytes induces failure of pre-implantation embryo development. This phenotype may be due to an essential function of the Hira complex in the zygote and/or during the following embryonic cleavages at the time around ZGA. Alternatively or additionally, the block in embryo development may be the result of defects that have arisen earlier during oocyte development. In this latter scenario, the genes encoding the Hira components would be considered maternal effect genes and the function of the complex would be required for the acquisition of developmental competence of mouse oocytes (Li et al., 2013). To distinguish between these two possibilities, we have analysed the transcriptional programmes in oocytes with a disrupted Hira complex (including Hira and Cabin1) during oocyte development. To precisely detect any earlier signs of aberrant transcriptomes, rather than collecting later stage oocytes (e.g. MII), we performed RNA sequencing (RNA-seq) in GV stage oocytes derived from both ZH and CabZ lines. A total of 1462 and 207 genes were significantly and differentially expressed (FDR adjusted *P*-value <0.05; fold change >2) in Hira and Cabin1 mutants compared with heterozygotes, respectively (Fig. 2A). Hira appeared to have a more-profound impact on the transcriptome than Cabin1 (888 of upregulated and 574 downregulated transcripts in the ZH mutants; 154 upregulated and 53 downregulated transcripts in the CabZ mutants; Fig. 2A). Nevertheless, we observed that there is a considerable similarity in the affected genes between ZH and CabZ mutants compared with heterozygous oocytes. Indeed, a high percentage of differentially expressed (DE) upregulated genes in CabZ mutants (57.5%; *P*<1.7E-70) overlap with upregulated genes in ZH mutants (Fig. 2B). This finding of co-regulation of genes by Hira and Cabin1 is consistent with observations in HeLa cells where separate knockdown of HIRA and CABIN1 produces similar effects (Rai et al., 2011). These data also suggest that both subunits of Hira complex (i.e. Hira and Cabin1) are equally important for the transcription outputs relevant for oocyte quality.

To address the possibility that the dysregulated genes were inherited from growing oocytes, we compared our datasets with a dataset generated by Ma et al. (2013), which extensively compared the transcriptomes of NSN (developmentally incompetent) to SN (developmentally competent) oocytes. Both ZH and CabZ regulated genes revealed minimal overlapping with genes expressed specifically at the NSN stage (Fig. S2A), indicating that the overall dysregulation of the genes of ZH and CabZ mutants is not due to a developmental arrest at an immature oocyte stage. In addition, we did not detect a significant change in the NSN/SN ratio in either ZH or CabZ oocytes after DNA dye staining [∼80% of SN in the ZH Het and KO oocytes (*n*>90 in each group, *P*=0.1); ∼85% of SN in the CabZ Het and KO oocytes (*n*>50 in each group, *P*=0.1)]. The morphological score indicated that the transition of NSN to SN in the ZH and CabZ GV oocytes appears to be normal.

We noted that, in general, both ZH (888 versus 574) and CabZ (154 vs 53) inactivation causes more of an increase than a decrease in transcription in GV oocytes (Fig. 2A). This finding prompted us to test whether there is a deficit in silencing global transcription in oocytes depleted of Hira complex (i.e. Hira and Cabin1). We focused on identifying a cohort of genes that are regulated by the Hira complex (i.e. Hira and Cabin1) and that may be directly associated with oocyte developmental competence. Gene ontology (GO) analyses showed that the upregulated genes following Hira deletion are involved in translation, apoptotic process and ribosomal biogenesis (Fig. 2C). Conversely, the downregulated genes are involved in DNA damage and repair, transcription, and chromatin remodelling (Fig. 2C). Notably, among the top-ranked GO terms from upregulated genes was an enrichment of a set of telomere-related genes, including zygotic genome activation-associated genes (e.g. *Zscan4c*, *Zscan4d* and *Zscan4f*) (Fig. 2C). As Zscan4 is also crucial for embryogenesis (Ko, 2016) and considered to be one of the two-cell-specific genes that is crucial for regulation of pluripotency/totipotency (Amano et al., 2013), we further compared our data with two-cell-specific gene datasets generated by Wu et al. (2016) [additional analyses by Percharde et al. (2018), Fig. S2B]. We found a significant overlap of two-cell-specific genes with both ZH- and CabZ-regulated genes (e.g. *Zscan4*, *Ddit3* and *Gm5039* with qRT-PCR validation in Fig. S2B; a similar aberrant upregulation effect was also observed in Ubn1 KD oocytes; Fig. S2B). This latter finding suggests that Hira inactivation may cause premature activation of transcription required for early embryo development. Although Zscan4 is regulated by Dux in early embryos (De Iaco et al., 2017), we did not detect *Dux* upregulation in the oocytes (Fig. S2C). The possibility that Dux regulates Zscan4 and other ZGA-related genes before early oocyte maturation seems unlikely given the very low-to-undetectable levels in GV oocytes (Fig. S2C). This analysis indicates that the depletion of Hira complex (i.e. Hira and Cabin1) in oocytes results in the ‘derepression’ of embryo-associated genes at earlier stages of development. In addition, our findings raise the possibility that the Hira complex may exert a repressive role to prevent precocious embryonic gene expression at the time of oocyte development that coincides with global transcription repression.

### Loss of function of the Hira complex causes a failure in the transcriptional silencing in fully grown oocytes

Recently, Ishiguro et al. (2017) described Zscan4 expression during the process of oogenesis, and noted that Zscan4 protein shows different staining patterns in NSN or SN types of GV oocytes (diffuse and stronger signals in NSN to spotty and weaker signals in SN). Additionally, the authors also observed that Zscan4 appearance correlates with the RNA Polymerase II-mediated transcriptional status during the NSN-to-SN transition. Thus, we hypothesised that the mis-upregulation of *Zscan4* and other genes observed in the ZH and CabZ mutant oocytes reflects an aberrant transcriptional status. To test this hypothesis, we used global or candidate approaches to confirm an increase in transcription.

**Fig. 3.**
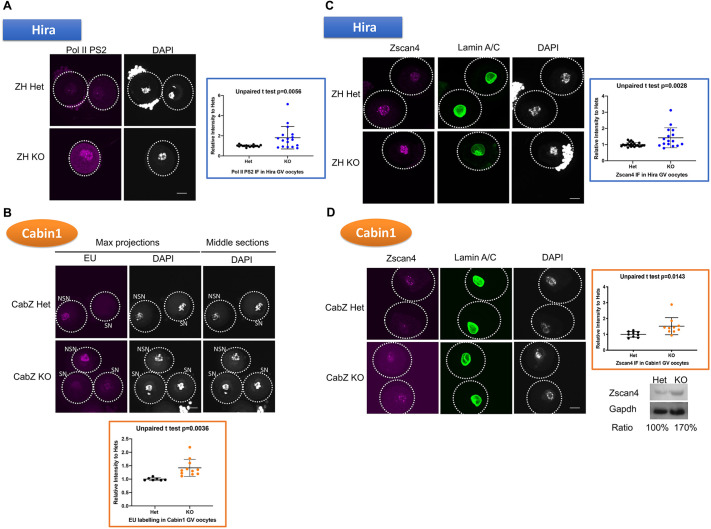
**Loss-of-Hira complex disrupts global transcription silencing and de-represses Zscan4 expression in GV oocytes.** (A) Hira mutant oocytes failed to silence global transcription. Immunofluorescent images (left panel) and quantification (right panel) of transcription status monitored by RNA polymerase II phosphorylated Ser2. (B) Cabin1 mutant oocytes failed to silence global transcription. Nascent RNA transcription was detected using an EU (5-ethynyl uridine) labelling assay. Upper panel, EU images; lower panel, quantification of EU (quantified SN oocytes only, see Materials and Methods). SN, non-surrounded nucleolus oocytes; NSN, non-surrounded nucleolus oocytes. (C) Zscan4 is upregulated in Hira mutant oocytes. Immunofluorescent images (left panel) and quantification (right panel) of Zscan4 and Lamin A/C of ZH KO and ZH Het oocytes. (D) Zscan4 is upregulated in Cabin1 mutant oocytes. Immunofluorescent images (left panel) and quantification (right panel), and western blot (lower right panel; *n*=3) of Zscan4 and Lamin A/C of CabZ KO and CabZ Het oocytes. Data are mean±s.d. Scale bars: 25 µm.

To monitor the global transcription status in Hira complex mutants (i.e. Hira and Cabin1), we performed immunofluorescence (IF) analysis of Pol II PS2, a marker of active transcription elongation, on ZH GV oocytes. Significant upregulation was observed in the ZH KO oocytes compared with ZH Het oocytes (180%, compared with ZH Het group of 100%; *P*=0.0056; Fig. 3A). We then applied 5-Ethynyl-uridine (EU) labelling to assess the nascent RNA synthesis in CabZ GV oocytes. We showed that the global level of transcription is also elevated in the CabZ KO oocyte (138%, compared with CabZ Het group of 100%; *P*=0.0036; Fig. 3B).

Using a candidate approach to confirm these aberrant transcriptions, we performed IF analysis to determine whether Zscan4 protein is indeed increased in ZH and CabZ oocytes. This strategy confirmed a significantly higher level of Zscan4 expression in both ZH KO (142%, compared with ZH Het group set at 100%; *P*=0.0028; Fig. 3C) and CabZ KO oocytes (151%, compared with CabZ Het group of 100%; *P*=0.0143; western blot also confirmed the upregulation ∼170%, compared with the CabZ Het group of 100%; Fig. 3D). We also noted that *Zscan4* along with other two-cell-expressed genes (e.g. *Ddit3* and *Gm5039* (*Eif1ad15*) persistently showed higher expression in both ZH or CabZ mutant MII stage oocytes (Fig. S3A). This result is in accordance with previously published datasets (Nashun et al., 2015) of Hira conditional mutant MII oocytes driven by both Zp3-Cre and Gdf9-Cre recombination approaches (Fig. S3B). These combined results support our hypothesis and demonstrate that the Hira complex is essential for the silencing of global transcription, one of the hallmark features of developmental competence in oocytes.

### H3K4me3 and H3K9me3 are not established efficiently in Hira and Cabin1 mutant GV oocytes

Transcription silencing in SN oocytes is associated with global chromatin remodelling (Andreu-Vieyra et al., 2010; Liu and Aoki, 2002; Zuccotti et al., 2002; Dumdie et al., 2018), another key feature of a competent oocyte (De La Fuente and Eppig, 2001). Recent research indicates that H3K9me2/3 and H3K4me3 are crucial for developmental competence (Dahl et al., 2016; Yeung et al., 2019; Zhang et al., 2016). Dumdie et al. (2018) have also demonstrated that H3K4me3 is involved in transcriptional quiescence in SN oocytes.

The Hira complex is responsible for H3.3 incorporation and H3.3 has been shown to be the only H3 histone subtype incorporated into fully grown oocytes (Nashun et al., 2015). Given the finding that H3.3 incorporation is reduced in Hira mutants (Lin et al., 2014), ZH KO, CabZ KO and Ubn1 knockdown oocytes (Smith et al., 2021), we sought to determine whether the pattern of histone marks is altered in ZH and CabZ mutant oocytes.

**Fig. 4.**
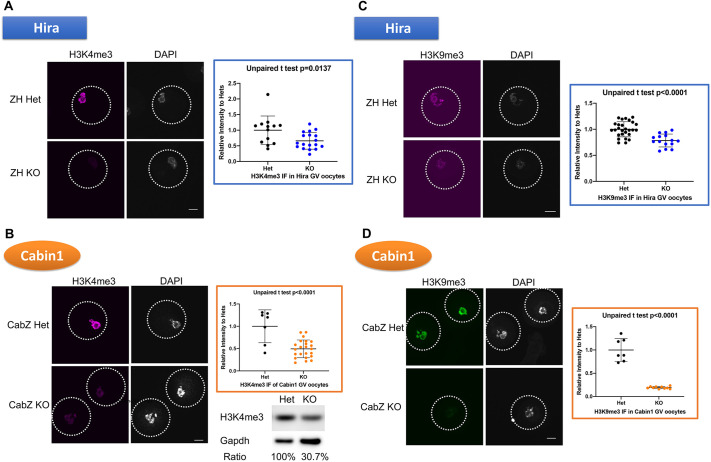
**The Hira complex is required to establish repressive histone marks in GV oocytes.** (A) Hira mutant oocytes fail to establish H3K4me3. Immunofluorescent images (left panel) and quantification (right panel) of H3K4me3 in ZH KO and ZH Het oocytes. (B) Cabin1 mutant GV oocytes fail to establish H3K4me3. Immunofluorescent images (left panel), quantification (upper right panel) and western blot (lower right panel) of H3K4me3 in CabZ KO and CabZ Het oocytes (*n*=3). (C) Hira mutant GV oocytes fail to establish H3K9me3. Immunofluorescent images (left panel) and quantification (right panel) of H3K9me3 in ZH KO and ZH Het oocytes. (D) Cabin1 mutant GV oocytes fail to establish H3K9me3. Immunofluorescent images (left panel) and quantification (right panel) of H3K9me3 in CabZ KO and CabZ Het oocytes. Data are mean±s.d. Scale bars: 25 µm.

Immunofluorescence of H3K4me3 shows decreased levels in ZH KO oocytes (66%, compared with ZH Het group of 100%; *P*=0.0137; Fig. 4A) as well as in CabZ KO oocytes (49%, compared with CabZ Het group of 100%; *P*<0.0001; Fig. 4B). Western blot analysis (Fig. 4B) confirmed these differences (∼31%, compared with CabZ Het group of 100%). Further analyses of the di-methyl group of H3K4, H3K4me2, also clearly showed lower levels of H3K4me2 in both ZH (43%, compared with ZH Het group of 100%; *P*<0.0001; Fig. S4A) and CabZ mutant oocytes (64%, compared with ZH Het group of 100%; *P*=0.0015; Fig. S4B). These data suggest that methylation of H3K4 is under the control of Hira-mediated H3.3 incorporation.

We next examined another panel of repressive histone modifications: those affecting methylation of H3K9. The level of H3K9me3 was reduced in both ZH KO (78%, compared with ZH Het group of 100%; *P*=0.0026; Fig. 4C) and CabZ KO oocytes (19%, compared with CabZ Het group of 100%; *P*<0.0001; Fig. 4D). The levels of H3K9me2 also revealed a substantial decrease in the ZH KO (50%, compared with ZH Het group of 100%; *P*<0.0001; Fig. S4C) and CabZ KO oocytes (79%, compared with ZH Het group of 100%; *P*=0.0022; Fig. S4D).

As shown in Fig. S4E,F, downregulation of the heterochromatin mark (HP1β) was observed in both ZH (50%, compared with CabZ Het group of 100%; *P*<0.0001; Fig. S4E) and CabZ KO oocytes (21%, compared with CabZ Het group of 100%; *P*<0.0001; Fig. S4F). These results strongly indicate that the loss of either Hira or Cabin1 in oocytes leads to reduced levels of repressive histone marks. Moreover, changes in the panH3 level (75%, compared with CabZ Het group of 100%; *P*<0.0001 by IF and western blot; Fig. S4G) and H3K27me3 (177%, compared with CabZ Het group of 100%; *P*<0.0001; Fig. S4H) but not H3K27ac (Fig. S4H) suggest that histone marks may be more broadly altered in the CabZ mutant oocytes (Fig. S4H).

### Cabin1 is required for the chromatin configuration in GV oocytes

**Fig. 5.**
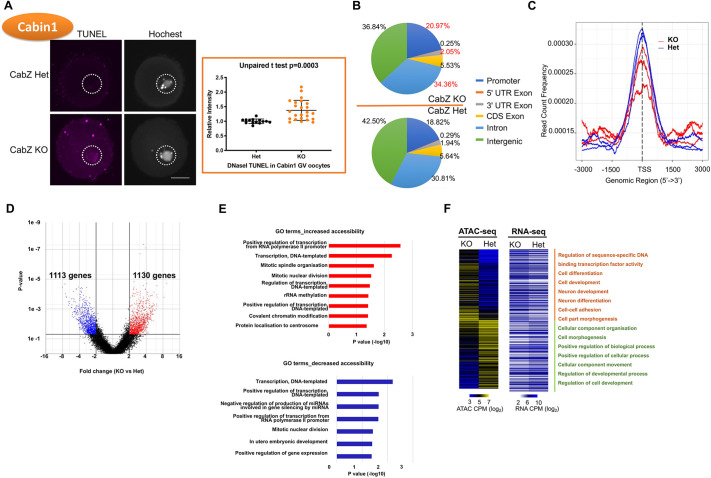
**Cabin1 is required to shape the condensed chromatin landscape in GV oocytes.** (A) Global chromatin accessibility is increased in Cabin1 mutant oocytes, as shown by DNaseI-TUNEL assay. Left panel: confocal images (left) and quantification (right) of DNaseI-TUNEL assay. Data are mean±s.d. Scale bar: 25 µm. (B) Pie charts represent the different distributions of ATAC-seq peaks in genome regions of CabZ Het and KO oocytes. (C) The enrichment of ATAC-seq peaks at annotated transcription start sites (TSS±3 kb) (normalised and on average) in Cabin 1 oocytes. The enrichment was measured at individual base pair level by FPM (fragments per million mapped reads). (D) Volcano plot shows the number of gene loci corresponding to increased (red) or decreased (blue) chromatin accessibility of genes in Cabin1 oocytes. (E) Top represented gene ontology terms for genes with consistent changes (increased, top; decreased, bottom) in chromatin accessibilities between CabZ KO and Het oocytes (≥4-fold change). (F) Heat map showing the identified genes with consistent changes in the chromatin accessibilities and their gene expression levels between CabZ Het and KO oocytes (*P*<0.05). CPM: counts per million reads mapped.

The loss of repressive histone marks in Hira and Cabin1 oocytes prompted us to further investigate the presence of possible alterations of chromatin structure in the CabZ mice. First, we examined the overall chromatin accessibility using a DNaseI-TUNEL assay (Nashun et al., 2015; Percharde et al., 2018) CabZ KO oocytes had significantly stronger TUNEL signals (146%, compared with CabZ Het group of 100%; *P*=0.0003; Fig. 5A), suggesting that the overall chromatin architecture had been converted to a less condensed status, echoing previous observations in Hira mutants (Nashun et al., 2015). These results also imply that the loss of key repressive histone marks closely reflects the perturbation of chromatin structure.

To further investigate the chromatin landscape of Hira complex mutant oocytes on a genome-wide scale, we performed an assay for transposase accessible chromatin using sequencing (ATAC-seq). Biological replicates of ATAC-seq showed highly reproducible results in CabZ Het and KO GV oocytes (Fig. S5A-D). The majority of ATAC-seq peaks in oocytes were detected in the intergenic and intron regions (Fig. 5B). ATAC-seq peaks were also preferentially enriched at the promoter, CDS exon and UTR exon regions (Fig. 5B), suggesting they might act as promoters and enhancers to regulate gene expression. Although overall chromatin is less accessible in CabZ KO oocytes compared with Het, as reflected by the majority of ATAC-seq peaks that are enriched in intergenic regions (Table S1, Fig. 5B), we found elevated ATAC-seq enrichment in regulatory regions in the CabZ KO oocytes, in particular in promoter (18.82% peaks in Het versus 20.97% in KO), 3′UTR (1.94% in Het versus 2.05% in KO) and intron (30.81% in Het versus 34.36% in KO) regions (Fig. 5B). The increased accessibilities were also generally correlated with increased levels of gene expression in these regions in the CabZ KO oocytes (Fig. S5E). Dynamic changes were also seen in close vicinity to the TSS sites (Fig. 5C). This ATAC-seq data supports the global alteration of chromatin landscape shown by DNaseI-TUNEL staining.

The chromatin accessibilities of specific genes or specific loci are dramatically different between CabZ KO and Het oocytes. We found the aberrantly increased and decreased chromatin accessibility between CabZ KO and Het oocytes corresponded to 1130 and 1113 gene loci, respectively (Fig. 5D). GO analysis revealed that the enriched genes are involved in transcription, mitosis, chromatin and development (Fig. 5E and Table S2). We performed integrated analysis using the ATAC-seq and the RNA-seq datasets, and identified 249 genes with consistent changes in their accessibilities and expression levels (Fig. 5F and Table S3). Genes with consistent change were involved in regulation of transcription factor binding, in cell functions that determine cellular components, and in differentiation and development (Fig. 5F).

In summary, we propose that Cabin1 (and Hira as an extension) modulates chromatin architecture by controlling the deposition of key repressive histone signatures and by maintenance of a globally condensed chromatin architecture. This is a hallmark feature of developmental competence in GV oocytes. Depletion of Cabin1 causes the reduction of repressive histone marks (i.e. H3K4me3 and H3K9me3) coupled with abnormal openness of accessible chromatin loci that leads to misregulation of maternal transcripts (e.g. *Zscan4*).

### Prolonged expression of Zscan4 results in loss of developmental competence and mimics the phenotype of two-cell embryos

**Fig. 6.**
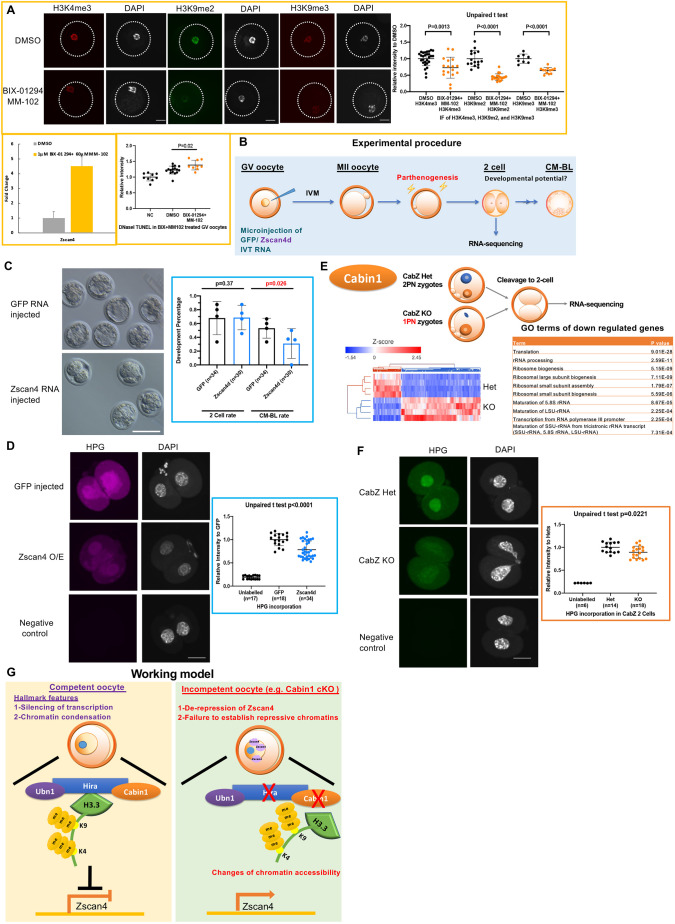
**Overexpression of Zscan4 impairs developmental competence.** (A) Histone methyltransferase inhibitor experiments confirmed that Zscan4 expression and overall chromatin accessibility are modulated by H3K4me3, H3K9me2 and H3K9me3 modifications. Oocytes were treated with BIX-01294 and MM-102 for 48 h. Immunofluorescence was used to confirm the inhibition of target histone marks. qRT-PCR was used to confirm the upregulation of Zscan4 after inhibitor treatment. A DNaseI-TUNEL assay was used to examine the global chromatin accessibility after BIX-01294 and MM-102 treatment. (B) Experimental procedure for overexpression of Zscan4 in oocytes and developmental/molecular analyses. (C) Zscan4d overexpressed oocytes compromised developmental potential. Left panel: developmental progression of GFP-injected and Zscan4-injected embryos at embryonic day 4 (E4). Right panel: quantification of two-cell cleavage and morula-to-blastocyst formation rate. (D) Global protein synthesis is repressed in Zscan4d overexpressed embryos by HPG incorporation assay. Left panel: HPG incorporation in Zscan4 overexpression and control (GFP injected and unlabelled) embryos. Right panel: quantification of HPG incorporation. (E) Gene ontology of RNA-seq result of Cabin1 mutant (CabZ KO) two-cell embryos showed that regulation of translation-related terms are among the top downregulated lists. (F) Global protein synthesis is repressed in the Cabin1 mutant (CabZ KO) two-cell embryos when assessed using a HPG incorporation assay. Left panel: images of HPG incorporation in CabZ KO and controls (CabZ Het and unlabelled) embryos. Right panel: quantification of the HPG incorporation assay. (G) A working model describes the mechanism controlling oocyte competence by Hira-mediated epigenome and transcriptome remodelling. Data are mean±s.d. Scale bars: 25 µm in A; 100 µm in C; 25 µm in D,F.

To clarify how histone marks regulate the derepression of Zscan4, we initially performed a series of pharmacological inhibitor experiments. We used (1) BIX-01294, an inhibitor of G9a histone lysine methyltransferase (Kubicek et al., 2007), which decreases the level of H3K9me2; (2) MM-102, an inhibitor for MLL1, the core complex of H3K4 histone lysine methyltransferase, which results in decreased levels of H3K4me3 (Karatas et al., 2013); and (3) the combined treatment of BIX-01294 with MM-102 (Fig. S6A and Fig. 6A). We cultured wild-type GV oocytes with these inhibitors to monitor whether the level of Zscan4 increases after withdrawal of repressive histone marks. We first confirmed the efficiency of the inhibitors. In treatment (1), the level of H3K9me2 was reduced in the BIX-0129-treated group as well as H3K9me3 levels (Fig. S6A); in treatment (2), the level of H3K4me3 was reduced in the MM-102 treated group (Fig. S6A); and the combined treatment (3) successfully induced a reduction in the levels of H3K9me2 (51%, compared with DMSO group of 100%; *P*<0.0001) and H3K9me3 (64%, compared with DMSO group of 100%; *P*<0.0001), and also reduced the level of H3K4me3 (73%, compared with DMSO group of 100%; *P*=0.0013; Fig. 6A). Using this pharmacological approach, we observed elevated expression of *Zscan4* by qRT-PCR after all three treatments (Fig. S6A and Fig. 6A) as well as increased chromatin accessibility (Fig. 6A). These results indicate that the repressive histone marks (i.e. H3K4me3 and H3K9me3) act as upstream regulators to modulate the proper expression of Zscan4.

To further determine whether misregulation of Zscan4 in the oocyte directly contributes to consequent developmental arrest, we forced the expression of Zscan4 in wild-type oocytes. We used the same strategy for Zscan4d RNA injection in wild-type GV oocytes as for our MO injections, followed by *in vitro* maturation, selection of MII oocytes for parthenogenetic activation and subsequent *in vitro* culture (Fig. 6B). Successful overexpression was validated by qRT-PCR (approximately fourfold compared with the uninjected oocyte group), IF (∼230% compared with uninjected group) and western blot (∼200% compared with uninjected group) of Zscan4 [Fig. S6B,C; the upregulation of Zscan4 was observed in CabZ and ZH mutant oocytes (between 140 and 170%; Fig. 3A,B)]. The developmental potential of the embryos was determined by the progression to morula-to-blastocyst stage embryos. There was no significant difference in the proportion achieving the two-cell cleavage rate between the Zscan4 overexpression group and the GFP-injected control. By contrast, the developmental rate to morula-to-blastocysts was significantly reduced in the Zscan4 overexpression group (31%, compared with GFP-injected group of 53%; *P*=0.026; Fig. 6C). However, very little change in key histone marks (H3.3, H3K4me3 and H3K9me3) and in chromatin accessibility was observed by IF and DNaseI-TUNEL assays, respectively, in the Zscan4-overexpressing oocytes (Fig. S6D). This last finding suggests that the alterations of key repressive histones in both ZH and CabZ mutants are not triggered by the upregulation of Zscan4. The mechanism of Zscan4 expression is more likely mediated by upstream pathways that rely on histone modifications. These results also provide evidence that timely expression of Zscan4 is crucial for embryogenesis.

Knockdown/knockout of maternal genes that are crucial for zygotic genome activation and early embryogenesis (e.g. Brg1, Mll2 and Kdm1a) often induces an overall reduction of initiation of translation (Bultman et al., 2006; Ancelin et al., 2016). Indeed, two-cell stage parthenogenetic embryos derived from Zscan4-overexpressing oocytes displayed a significant reduction of global protein synthesis compared with two-cell stage embryos from GFP RNA-injected oocytes, as assessed by the homopropargylglycine (HPG) incorporation assay (73%, compared with GFP group of 100%; *P*<0.0001; Fig. 6D).

Intriguingly, comparison of RNA-seq data from the CabZ KO and CabZ Het 2-cells revealed that top-ranked GO terms of DE downregulated genes appeared to be classified as translation, including rRNA processing, ribosome assembly and ribosome biogenesis (Fig. 6E). HPG incorporation assays also showed a significantly lower protein production in the CabZ KO compared with CabZ Hets two-cell embryos (89%, compared to CabZ Het group of 100%; *P*<0.0221; Fig. 6F), which agrees with the Zscan4 overexpression phenotype. The consistency of impairment of protein production in CabZ mutants and Zscan4 overexpression two-cell embryos indicates that there is a mutual deficit in the initiation of ZGA.

Moreover, RNA-seq analysis of Zscan4-overexpressing two-cell embryos revealed that transcripts involved in ‘regulation of transcription’, ‘blastocyst development’ and ‘ribosome small subunit assembly’ were overrepresented among the downregulated GO terms (Fig. S6E). Above all, Rps19, an essential ribosomal protein that is crucial for blastocyst development (Matsson et al., 2004) was shared in downregulated genes of CabZ and Zscan4-overexpressing two-cell embryos. qRT-PCR also confirmed the downregulation of *Rps19* in agreement with the results of RNA-seq (Fig. S6F). Therefore, likely failure to begin ZGA associated with the decline of overall protein production in the CabZ KO two-cell embryos is likely due to the derepression of Zscan4 in oocytes. This, in turn, hampers embryonic development.

To address whether the abnormally elevated level of Zscan4 in oocytes (Fig. 3A,B) in Hira/Cabin1 mutants is responsible for the loss of developmental competence, we performed a rescue experiment. Partial knockdown of Zscan4 was achieved by microinjection of Zscan4 morpholinos (MO) (knockdown validation in Fig. S7A). Zscan4 MO and control (Ctrl) MO were microinjected into CabZ KO GV oocytes and the phenotypes were compared with uninjected and Ctrl MO injected CabZ Het GV oocytes. Following maturation to MII, oocytes were parthenogenetically activated and their developmental progression monitored. We observed that CabZ Het oocytes (both uninjected and Ctrl MO injected) can successfully develop to morula-to-blastocyst stage. By contrast, as stated above, CabZ KO GV oocytes (injected with Ctrl MO) revealed impaired developmental potential by showing a very low propensity of embryos reaching two cells and/or developing beyond the two-cell stage. Importantly, with downregulation of Zscan4 on a CabZ KO background, embryo development is partially restored (Fig. S7B). This rescue experiment further supports the observation that repression of Zscan4 in the oocyte is crucial for oocyte-to-embryo transition.

Taken together, our findings can be accommodated in a model (Fig. 6F) whereby competent oocytes display two hallmark features of (1) silencing of transcription and (2) regulation of overall chromatin condensation by the Hira complex mediated by H3.3 incorporation. This state is associated with repressive marks of H3K4me3 and H3K9me3 that suppress activation of a subset of early embryonic genes (e.g. *Zscan4*). Conversely, incompetent oocytes, such as those resulting from maternal disruption of Hira or Cabin1, fail to establish histone marks and H3.3 incorporation, as well as K4me3 and K9me3 modifications. As a consequence, an aberrant chromatin landscape results in premature activation of gene expression (e.g. derepression of Zscan4 and other embryonic two-cell genes) eventually leading to a developmental arrest.

## DISCUSSION

Using complementary approaches, we demonstrate that the Hira-complex plays an essential role in the development of oocyte competence to sustain embryo development. This complex is involved in shaping chromatin by promoting incorporation of H3.3 enriched with H3K4me3 and H3K9me3 marks in fully grown quiescent oocytes. The methylated H3.3 reduces chromatin accessibility and represses transcription of two-cell genes, including *Zscan4*, *Ddit3* and *Gm5039*, genes with crucial functions later in embryonic development. Failure to repress these genes causes delayed effects after fertilisation/parthenogenesis in the embryo. Consistent with this conclusion, overexpression of Zscan4 prevents the proper initiation of zygotic genome activation and causes a developmental arrest that recapitulates the key phenotypes of loss-of-function of Hira-complex mutants. A rescue of the Cabin1 phenotype by downregulation of Zscan4 on a Cabin1 null background reinforces this conclusion. Thus, enforcing a repression of transcription in quiescent GV oocytes is an indispensable step towards preparation for the programmed changes in gene expression in the zygote that are crucial for establishing totipotency.

Our data provide insight into the molecular mechanism involved in this generalised repression of transcription enforced in the GV oocyte prior to resumption of meiosis. Cabin1 has been identified as a vertebrate-specific subunit of Hira complex (there is no Cabin1 homolog in fly). Here, we have identified Cabin1 as a new maternal factor that is crucial for fecundity. Cabin1 homozygous null mutants exhibiting embryonic lethality during organogenesis have been reported (Esau et al., 2001). Cabin1 roles not only include binding to calcineurin and myocyte enhancer factor 2 (MEF2) in T-cell development, but also chaperoning H3.3 incorporation via binding to trimerised Hira (Ray-Gallet et al., 2018). The embryonic lethality phenotype in Cabin1 mutants likely results from insufficient incorporation of H3.3. Moreover, it has been shown that Cabin1 recruits Suv39H1, a major H3-K9 methyltransferase required to repress myocyte enhancer factor 2 transcription activity in T cells (Jang et al., 2007). In agreement with this view, we also observed a decreased level of H3K9me2/3 and HP1 in Cabin1 mutant oocytes. This finding supports the notion that Cabin1 likely has a similar mechanism of action in oocytes to suppress the developmental genes programmed to be expressed at later stages. Similarly, Cabin1 may play a function in the PRC1/PRC2- or Suv39h1/Suv39h2-mediated maternal-to-zygotic transition (Nestorov et al., 2015; Banaszynski et al., 2013).

Despite identification years ago of two distinctive features of competent oocytes, chromosomal condensation and formation of heterochromatin rims (Zuccotti et al., 2002; Debey et al., 1993), as well as transcriptional silencing (Bouniol-Baly et al., 1999), the underlying regulatory mechanisms repressing transcription have remained elusive. With our study, we demonstrate that H3K4me3/H3K9me3 are decreased in Hira and Cabin1 mutant oocytes. The observed decrease of H3K4me3 in the Cabin1 mutant oocytes suggests that the compromised accumulation of H3K4me3 (possibly the non-canonical form) has a repressive role in oocytes. The large number of upregulated genes that follow Hira inactivation is consistent with this view. Although H3K4me3 is widely accepted to be an active transcription mark (i.e. there is a high positive correlation between the transcriptome and canonical H3K4me3 incorporation), an additional role has been proposed for it specifically in the oocyte [i.e. non-canonical H3K4me3, which not only shows a lower correlation with transcription but also may play a role in genome silencing during oogenesis (Zhang et al., 2016)]. In addition, Zhang et al. (2016) and Dumdie et al. (2018) both documented that removal of non-canonical H3K4me3 by either overexpression of the responsible enzyme, Kdm5b, or by depletion of the upstream regulator, Zfp36l2, induced reactivation of transcription in oocytes. These lines of evidence support that H3K4me3 acts in a repressive role, particularly during oogenesis.

Although MLL2 (Kmt2d) has been reported as one of the important H3K4 methyltransferases in the oocyte (Andreu-Vieyra et al., 2010; Hanna et al., 2018), the role of other MLL proteins has not been extensively studied. Our result revealed that MM-102 (a MLL1 inhibitor) induced the reduction of H3K4me3, which supports the potential regulatory role of MLL1 in oocytes; however, further studies need to be conducted before drawing any conclusion. Therefore, future effort should focus on the extensive profiling of H3K4me3 and the other histone marks in Cabin1 mutants to pinpoint the genome-wide crucial transcriptional regulators.

The Hira-complex plays an essential role in remodelling of chromatin at a crucial transition of oocyte development. The altered H3.3 methylation that follows disruption of the Hira complex is associated with aberrant chromatin composition, as indicated by ATAC-seq and altered transcription measured by RNA-seq, with most affected genes being upregulated. These observations strongly argue that the repressive H3.3 methylation marks are indispensable for inducing the transcription silencing observed at this stage. The overall aberrant chromatin landscape in Cabin1 mutant oocytes could also be a consequence of the failure of nucleosome replacement of H3.3 mediated by the Hira complex (Schneiderman et al., 2012), which leads to the increase in chromatin accessibility (Navarro et al., 2020). Misregulation of transcription may result from the changes of chromatin accessibility (as observed in the ATAC-seq result) regulated by Hira directly via reduced nucleosome replacement (van der Heijden et al., 2007) or indirectly by H3.3 affecting associated histone modifications (e.g. H3K4me3 and H3K9me3) or DNA methylation (Nashun et al., 2015).

Failure of incorporation of methylated H3.3 in the oocyte leads to an early embryo arrest, indicating a loss of competence to develop as embryos. Our data, as well as those of others, is inconsistent with the idea proposed by Zuccotti et al. (2002) that the heterochromatin rim can be used as the unequivocal selection marker for competent oocytes. Both Hira and Cabin1 mutant oocytes have lost their developmental competence despite maintaining the heterochromatin rim structure. It is likely that, in addition to forming the heterochromatin rim structure, additional mechanisms in the oocyte are necessary for a higher degree of chromatin condensation to complete the final stage of the NSN-SN transition. According to this view, oocyte repressive histone modifications, including H3K4me3 and H3K9me3, may serve as the ‘competent oocyte’ histone codes deposited by the Hira complex. Notwithstanding Xia et al. (2019) reporting of the absence of ‘non-canonical’ H3K4me3 in human oocytes, the global transcriptional silencing event did take place during NSN-SN transition. Genome-wide profiling by Hi-C (Gassler et al., 2018) and/or in combination with a higher resolution imaging technique such as electron spectroscopic imaging (ESI) (Ahmed et al., 2010) should add further support to this idea.

Zscan4, a unique two-cell-specific transcription factor, plays pivotal roles during development (Falco et al., 2007), cell fate fidelity (Amano et al., 2013), regulation of transcription (Hung et al., 2013; Zhang et al., 2019), telomere and genomic stability (Zalzman et al., 2010), and is a specific marker of two-cell-like embryonic stem cells (Zalzman et al., 2010; Rodriguez-Terrones et al., 2018). In addition, misregulation of Zscan4 accompanies failure of progression through embryo development (Percharde et al., 2018; Matoba et al., 2014; Dan et al., 2014) and an imbalance of protein synthesis (Hung et al., 2013). In agreement with these findings, overexpression of Zscan4 in GV oocytes compromises oocyte developmental potential and mimics the key phenotypes of Cabin1/Hira mutants. In both cases, advanced developmental stages of the embryo are affected, owing to the impairment of ZGA initiation and protein production.

Intriguingly, embryonic Zscan4 expression is under tight control by chromatin regulators such as the PRC1 complex (i.e. Ring1/Rnf2; Posfai et al., 2012) and methylation of H3K9 (Matoba et al., 2014). Ring1/Rnf2-deficiency in oocytes leads to the precocious expression of *Zscan4* and of lineage markers such as *Sox2*, *Klf4*, and *Eomes*, which are thought to be expressed in later embryonic stages (Posfai et al., 2012). We found that the BIX-01294 and MM-102 inhibitors also induced upregulation of *Zscan4* in oocytes, suggesting that *Zscan4* expression is downstream of the regulation of H3K4 and H3K9 by methylation. On the other hand, overexpression of Zscan4 did not alter histone marks or chromatin accessibility (Fig. S6D), supporting the notion that it acts downstream of these histone modifications. All these findings taken together provide a strong link between the H3.3, the Hira complex and the mechanisms of gene silencing occurring at this stage of oocyte development.

In summary, we demonstrate that the H3.3 chaperone Hira complex is essential for oocyte developmental competence by shaping its epigenome and its transcriptome. The oocyte suppresses its totipotent/pluripotent potential by repressing a subset of early embryonic genes via deposition of repressive histones. Even though the chromatin condensation process is conserved in most mammalian oocytes, diverse dynamics have been noted. For example, four stages of chromatin condensation have been observed in bovine oocytes (Luciano et al., 2014). The properties shared between bovine and human oocytes should prompt a re-evaluation of the levels of H3K4me3, H3K9me3 and also Zscan4 in these species. Moreover, further research will be crucial to identify the Zscan4 targets and their involvement in regulation of chromatin condensation and the development of epigenetic biomarkers for competent oocytes. These, in turn, may provide new strategies to improve assisted reproductive technologies.

## MATERIALS AND METHODS

### Animals

Mouse experiments were approved by the University of Edinburgh's Animal Welfare and Ethical Review Board (AWERB) and carried out under the authority of a UK Home Office Project Licence. C57BL/6 wild-type mice were purchased from Charles River Laboratories. The mouse lines used in this study carried conditional floxed alleles for Cabin1 and for Hira on C57BL/6 backgrounds (both were provided by Prof. Peter Adams previously at the Beatson Institute, UK, now relocated to Sanford Burnham Prebys, La Jolla, CA, USA). Floxed Cabin1 (Cabin1^fl/fl^) and floxed Hira (Hira^fl/fl^) mouse lines were crossed with a Zp3-cre mouse line (de Vries et al., 2000) provided by Prof. Petra Hajkova (Imperial College London, UK) that expresses cre recombinase in the female germline to generate heterozygous and homozygous mutant oocytes.

### Oocyte/embryo culture and micromanipulation

Female mice were superovulated by administration of 7.5 IU pregnant mare serum gonadotrophin (PMSG from Prospec Protein Services) and 48 h later fully grown oocytes were isolated. Oocytes were cultured in M16 medium at 37°C, 6% CO_2_ and 5% O_2_. Parthenogenetic activation was carried out as previously described (Lin et al., 2014), matured MII oocytes were cultured in calcium-free CZB medium with 10 mM SrCl_2_ for 5 h.

For zygote collection, 48 h after PMSG administration 7.5 IU human chorionic gonadotrophin (hCG, Chorulon from Intervet) was injected and the mice were mated with C57BL/6 males. The following day, zygotes were collected from the oviducts. Embryos were cultured in KSOM medium at 37°C, 6% CO_2_ and 5% O_2_.

Micromanipulation was performed as described previously (Lin et al., 2013, 2014). Micromanipulation platform was equipped with a microinjector (FemtoJet 4i, Eppendorf), an inverted microscope (Leica, DMi8) and micromanipulators (Narishige). For Ubn1 knockdown experiments, oocytes were microinjected with control (Gene Tools) or Ubn1 antisense morpholino oligos (CTCCGACATGGCTACCAACAAGTCT). For H3.3 knockdown experiments, morpholinos were identical to those in our previous paper (Lin et al., 2013). Zscan4 antisense morpholino oligos (TGCCTGCTGTGAAGCCATTGT) were used for rescue experiment. For Zscan4 overexpression experiments, Zscan4 (Addgene 61831) and GFP (a gift from M. Anger, Institute of Animal Physiology and Genetics CAS, Czech Republic) *in vitro* transcription RNA were carried out using mMessage mMachine kit (Life Technologies) and a poly(A) tail was added (Applied Biosystems AM1350). Unincorporated nucleotides were then removed with a RNA clean up kit (Zymo R1015).

### Immunofluorescence

Immunofluorescence (IF) experiments were performed as previously reported (Lin et al., 2013, 2014). Images of stained oocytes/embryos were acquired by a spinning disk confocal (CSU-W1, Yokogawa) on an upright microscope frame (BX-63, Olympus) using a 30 or 60× silicon oil immersion objective (UPLSAPO 60XS2, Olympus). IF signal intensity was quantified using Fiji software. For quantifying the IF intensity of nuclear proteins in the oocytes, we only selected the nuclear region of interest for quantification and only selected ‘surrounded nucleolus, SN oocytes in which chromatin is condensed and forms a ring structure surrounding the nucleolus after DNA dye staining’ oocytes for comparison between groups. Antibodies used are listed in Table S4.

### Western blotting

Oocytes were washed in PBS and frozen at −80°C. Thirty oocytes were lysed in Reducing SDS loading buffer and denatured for 5 min. Proteins were separated by gradient precast 4-12% SDS-PAGE gel (Thermo Fisher) and transferred to Immobilon P membrane (Millipore) by the semi-dry blotting system. Membranes were blocked by 5% skimmed milk for 1 h and incubated with H3K4me3 (Diagenode, 1541003; 1:500), H3 (Abcam, ab1791; 1:500), Zscan4 (Abcam, 97748; 1:250) and Gapdh (Santa Cruz Biotechnology, 97166; 1:500) primary antibodies diluted in 1% milk*/*TTBS overnight. The membranes were incubated in peroxidase donkey anti-rabbit secondary antibody (Jackson ImmunoResearch, 711-035-152, 1:10,000) for 1 h at room temperature. Proteins were visualised by chemiluminescence using ECL (Amersham), and imaged and quantified using an Azure 600 Imager.

### Inhibitor experiments

GV oocytes from wild-type mice were cultured in M16 medium containing IBMX (both Sigma) at 37°C, 6% CO_2_ and 5% O_2_, and supplemented with: BIX-10294 (Stratech) at final concentrations of 0.1 µM and 1 µM (Huang et al., 2017); MM-102 (Cayman Chemicals) at final concentrations of 6 µM or 60 µM, within the range previously reported (Zhang et al., 2018); or a combination of 1 µM BIX-01294 and 60 µM MM-102. The inhibitors were dissolved in DMSO (Sigma) and an equivalent concentration was used for the control groups. After 48 h the surviving GV oocytes were either fixed in 4% paraformaldehyde (Thermo Scientific) for investigation by IF, or frozen at −80°C for RNA isolation and subsequent qRT-PCR.

### Nascent transcription and translation assays

For nascent transcription assays, oocytes were incubated in M16 medium with IBMX supplemented with 1 mM EU (5-ethynyl uridine) for 2 h, fixed in methanol, then a Click-iT RNA Imaging kit (Thermo Fisher Scientific) was used and visualisation was by confocal microscopy. Changes in protein expression were monitored using a Click-iT Protein Synthesis Assay kit (Thermo Fisher Scientific). Oocytes and two-cell embryos were cultured in M16 medium with the methionine analogue HPG at a dilution of 1:500. Newly synthesised proteins incorporating the HPG were visualised by confocal microscopy.

### RNA isolation and qRT-PCR

RNA was extracted from oocytes/embryos using PicoPure RNA isolation kit (Arcturus) and cDNA made using iScript cDNA Synthesis kit (BioRad). Brilliant III SYBR green QPCR Master Mix (Agilent Technologies) was used for qRT-PCR and reactions run on a Roche LightCycler 96 real-time PCR system. mRNA levels of genes of interest were calculated by comparison with Hprt1 and H2A mRNA levels using REST software. Primers used are listed in Table S5.

### RNA-seq and data analysis

Ten oocytes collected from each experimental treatment or control were pooled and cDNA was amplified using Smart-seq2 v4 kit with minor modifications from manufacturer's instructions (four replicates of each for both Hira and Cabin1 Het and KO oocytes, three replicates for Zscan4 overexpression and four replicates with GFP controls). Briefly, oocytes were lysed, and mRNA was captured and amplified with the Smart-seq2 v4 kit (Clontech) following the manufacturer's instructions. RNA-seq libraries were constructed using Nextera XT DNA Library Preparation Kit (Illumina) and multiplexed using Nextera XT Indexes kit (Illumina) according to the manufacturer's instructions. Libraries were quantified by Qubit (Life Technologies) and Tapestation 4200 (Agilent). Pair-end 150 bp sequencing was performed on an Illumina HiSeq X sequencer.

Multiplexed sequencing reads that passed filters were trimmed to remove low-quality reads and adaptors by TrimGalore-0.4.3. Clean reads were aligned to the mouse genome (GRCm38/mm10) by using STAR with default parameters. Approximately 30 million reads per individual cell were generated. Individual mapped reads were adjusted to provide FPKM (fragments per kilobase of exon model per million mapped fragments) values with RefSeq genes as reference. Differential gene expression analysis was performed by a Partek Flow GSA algorithm with default parameters. The genes were deemed differentially expressed if they provided a false discovery rate (FDR) adjusted *P*<0.05 and fold change >2. Gene Ontology (GO) and pathways analysis was performed using IPA (Ingenuity Pathway Analysis), with an FDR adjusted *P*≤0.05 deemed statistically significant.

### ATAC-sequencing and data analysis

The ATAC-seq libraries of oocytes were prepared as previously described with minor modifications (Wu et al., 2016, 2018). Briefly, approximately 100 pooled oocytes (three replicates of Cabin1 Het and KO GV oocytes) were lysed in ice-cold lysis buffer [10 mM Tris-HCl (pH 7.4), 10 mM NaCl, 3 mM MgCl_2_ and NP-40 (0.5%)] for 15 min on ice to prepare the nuclei. Nuclei were then incubated with the Tn5 transposase (TDE1, Illumina) and tagmentation buffer at 37°C for 30 min with shaking on a thermomixer at 500 ***g***. Tagmentated DNA was purified using MinElute Reaction Cleanup Kit (Qiagen). PCR was performed to amplify the ATAC-seq libraries using Illumina TrueSeq primers and multiplex by indexed primers under the following PCR conditions: 72°C for 3 min; 98°C for 30 s; 15 cycles of 98°C for 15 s, 60°C for 30 s and 72°C for 3 min; followed by 72°C for 5 min. The ATAC-seq libraries were purified twice with the 1.1× AMPure beads (Beckman) and quantified by Qubit (Life Technologies) and Tapestation 4200 (Agilent). ATAC-seq libraries were sequenced on the Illumina HiSeq X platform with 150 bp end reads.

All quality assessed ATAC-seq reads were aligned to the mouse reference genome using Bowtie 2.3 with following options: --very-sensitive -X 2000 --no-mixed --no-discordant. Alignments resulted from PCR duplicates or locations in mitochondria were excluded. Only unique alignments within each sample were retained for subsequent analysis. Principal component analysis (PCA) was performed on pairwise correlation data in R with functions prcomp. ATAC-seq peaks were called separately for each sample by MACS2 (Zhang et al., 2008) with following options: --keep-dup all –nolambda --nomodel. The ATAC-seq bigwig files were generated using bamcoverage from deeptools. The ATAC-seq signals were visualised in the Integrative Genome Viewer genome browser. The reproducibility between ATAC-seq libraries were assessed using PCA, hierarchical clustering analysis (Partek algorithm) and scatter plot analysis (using Pearson's r coefficient, which was calculated using ‘cor’ function and were visualised using ‘smoothScatter’ function available in R). Peaks in individual samples from the same development stage were subsequently merged using bedtools (https://bedtools.readthedocs.io/en/latest/). The annotations of genomic features, including transcription start sites (Matsson et al., 2004), transcription end sites (TES), promoters, CDS, introns, 5′ UTR, 3′ UTR and intergenic regions were downloaded from UCSC genome browser. Promoters were defined as 500 bp upstream and downstream of the TSS for each annotated gene (TSS±500 bp). Intergenic regions were defined as genomic regions before the TSS of the first gene and after the TES of the last gene in each chromosome, and in between the TES and TSS of two consecutive genes.

For downstream analysis, we normalised the read counts by computing counts scaled by the number of sequenced fragments multiplied by one million (CPM). Chromatin accessibility in each developmental stage was assessed by the number of ATAC-seq fragments (FPM) mapped to the defined gene region in all samples from that stage. Integrative analysis on the changes of chromatin accessibilities and their corresponding gene expression changes between treatment groups was conducted using the genes with differential accessibilities (two-fold change threshold) and the differential expressed genes (FDR adjusted *P*<0.05 and fold change >2). Gene ontology (GO) and pathways analysis was performed using IPA, with an FDR adjusted *P*≤0.05 deemed statistically significant.

### DNaseI-TUNEL assay

Oocytes were permeabilised by 0.5% Triton X-100 in pre-extraction buffer (300 mM sucrose, 25 mM HEPES, 1 M CaCl_2_, 50 mM NaCl and 3 mM MgCl_2_) for 5 min before digesting with 0.2 U/ml of DNase I (Tanaka et al., 2001). Embryos were then fixed in 4% PFA. TUNEL assays (Click-iT TUNEL Imaging assay, Thermo Fisher Scientific) were carried out according to manufacturer's instructions. Images were visualised by confocal microscopy.

### Statistical analyses

For analyses of the percentage of oocyte maturation and embryonic development, the χ^2^ test was used as previously described (Lin et al., 2013, 2014). For analyses of IF intensity, signals of EU, DNaseI-TUNEL and HPG incorporation, two-tailed *t*-test with unequal variance was used. All error bars indicate s.d.

## Supplementary Material

Supplementary information

Reviewer comments
