# Supplementary material for: The H3.3 chaperone Hira complex orchestrates oocyte developmental competence
Source: Development. 2022 Feb 28;149(5):dev200044. doi: 10.1242/dev.200044 (PMC8959146; doi:10.1242/dev.200044)
Supplement: Supplementary information [file develop-149-200044-s1.pdf]

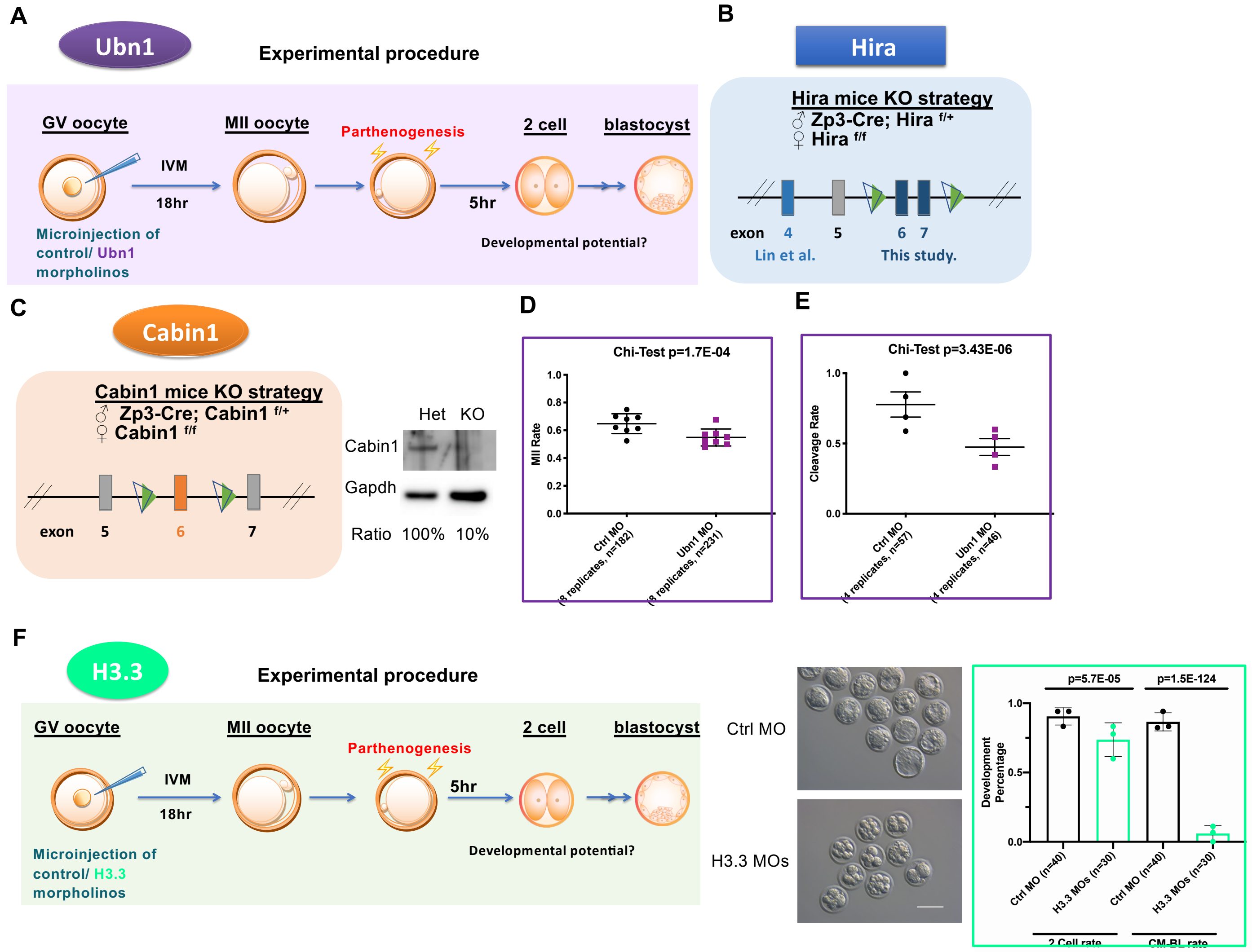

Fig. S1. Experimental approaches for investigation of the maternal role of the Hira complex.(A) Ubn1 knockdown and developmental assay. GV oocytes were microinjected with Ubn1 and control antisense morpholino oligos. Injected oocytes were matured *in vitro*, parthenoactivatedby $SrCl_2$for 5 hours and cultured *in vitro*. 2-cell formation and blastocyst development potential were monitored.

(B) Strategy of generation of knockout Hira in oocytes. Exon 6 and 7 regions (this study; (Rai et al., 2014)) of Hira were deleted in the oocyte using Zp3-cre recombination approach. Previous paper published by us (Lin et al., 2014) where we deleted the exon 4 region.

(C) Strategy of generation of knockout Cabin1 in oocytes. Upper panel: Exon 4 region of Cabin1 was deleted in oocytes using Zp3-cre recombination approach. Right panel: western blot validation of Cabin1 mutant (CabZ KO) and heterozygous control (CabZ Het) oocytes (n=2).

(D) Knocking down of Ubn1 in the oocyte impaired oocyte maturation potential. MII oocyte maturation rate was monitored in Ubn1 knockdown oocytes.

(E) Knocking down of Ubn1 in the oocyte impaired embryo cleavage. 2-cell formation rate was monitored in Ubn1 knockdown oocytes.

(F) Maternal H3.3 is required for preimplantation development of parthenogenetically activated embryos. Upper panel: H3.3 knockdown approach was similar to Ubn1 knockdown. Lower panel: 2-cell formation and morula-to-blastocyst rate of H3.3 knockdown embryos was impaired. Scale bar: 100mm.

Development • Supplementary information

A

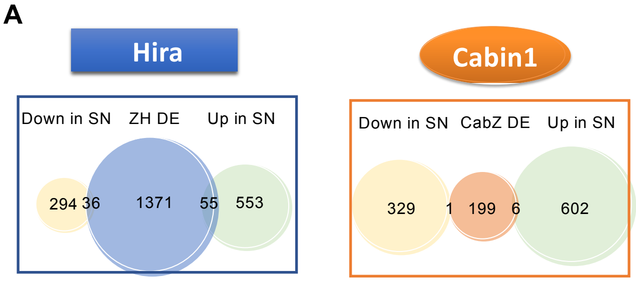

B

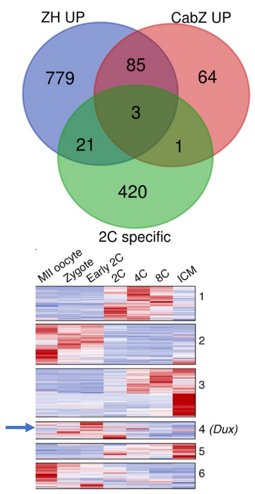

Percharde et al., 2018

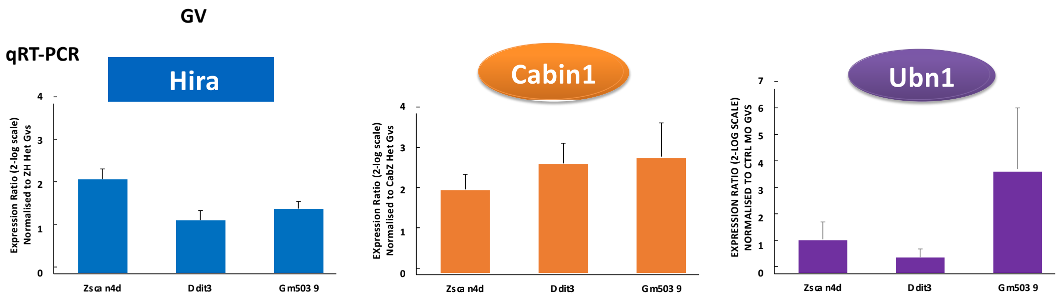

RNA-seq FPKM value

	Zscan4d	Ddit3	Gm5039
ZH Het	1.819	11.735	0.323
	3.498	11.606	0.469
	2.476	15.004	0.968
	2.535	16.976	0.955
ZH KO	33.904	181.380	41.300
	42.282	127.464	36.075
	80.803	53.372	16.328
	78.913	188.800	45.744

	Zscan4d	Ddit3	Gm5039
CabZ Het	0.061	0.258	0.028
	0.038	0.265	0.025
	0.057	0.211	0.025
	0.041	0.207	0.034
CabZ KO	0.104	0.531	0.317
	0.110	0.619	0.214
	0.096	0.491	0.198
	0.214	0.476	0.189

C **RNA-seq FPKM value**

	AW822073 (Duxf1)	Gm10807 (Duxf2)	Dux (Duxf3)	Gm4981 (Duxf4)	Gm19459 (Duxf5)
ZH Het	0.070	0.010	0.047	10.795	0.010
	0.010	0.206	0.010	14.043	0.010
	0.010	0.086	0.010	14.812	0.010
	0.010	0.134	0.010	10.650	0.057
ZH KO	0.010	0.010	0.081	5.405	0.132
	0.169	0.088	0.033	13.484	0.069
	0.010	0.200	0.038	4.481	0.153
	0.148	0.010	0.236	6.536	0.080

	AW822073 (Duxf1)	Gm10807 (Duxf2)	Dux (Duxf3)	Gm4981 (Duxf4)	Gm19459 (Duxf5)
CabZ Het	ND	ND	ND	0.425	ND
	ND	ND	ND	0.395	ND
	ND	ND	ND	0.346	ND
	ND	ND	ND	0.252	ND
CabZ KO	ND	ND	ND	0.352	ND
	ND	ND	ND	0.206	ND
	ND	ND	ND	0.283	ND
	ND	ND	ND	0.249	ND

Fig. S2. Bioinformatic analyses of RNA-seq results of Hira and Cabin1 mutant GV oocytes.

(A) Minimal overlapping of genes from either Hira or Cabin1 differentially expressed genes within growing oocytes (surrounded-nucleus, SN).
(B) A subset of 2 cell specific genes derepressed in both Hira and Cabin1 mutant GV oocytes. Left panels: Venn-diagram shows the overlapping genes which are upregulated both in the Hira and Cabin1 mutant (ZH KO and Cab KO) GV oocytes. Upper right panel: qRT-PCR confirmed the selected upregulated genes, Zscan4d, Gm5039, and Ddit3, in both ZH and CabZ KO mutant GV oocytes and Ubn1 knockdown GV oocytes. Lower right panels: FPKM values of RNA-seq of Hira and Cabin1 GV oocytes respectively.
(C) Expression of Dux gene family in Hira and Cabin1 GV oocytes. Tables list the FPKM values from of Hira and Cabin1 GV RNA-seq datasets. ND: not detected.

Development • Supplementary information

A

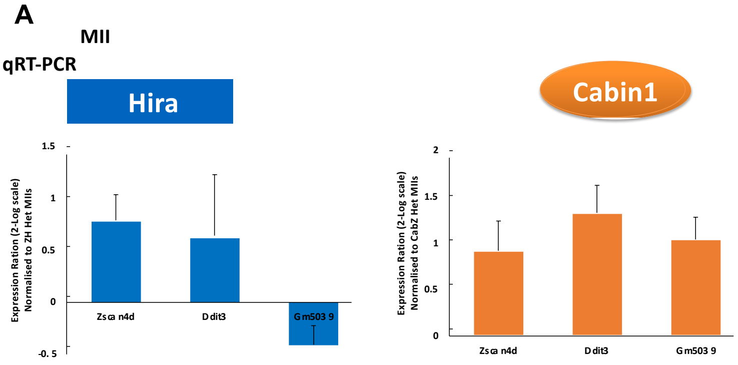

B

logFC	Gdf9-Cre Hira KO	Zp3-Cre Hira KO
Zscan4c	4.97	1.38
Zscan4d	4.43	2.18
Zscan4f	4	2.29
Ddit3	2.39	0.465
Gm5039	NA	NA

RNA seq result by Nashun et al.

Fig. S3. Zscan4 is upregulated in Hira and Cabin1 mutant MII oocytes and 1 cell embryos.

(A) Zscan4 is upregulated in both Hira and Cabin1 mutant MII oocytes. qRT-PCR of Zscan4, Ddit3, and Gm5039 expression in ZH and CabZ KO MII oocytes. Expression was normalised to ZH Het and CabZ Het controls respectively.

(B) List of Zscan4 fold changes of Hira mutant MII oocytes from previously published dataset (Nashun et al., 2015).

Development • Supplementary information

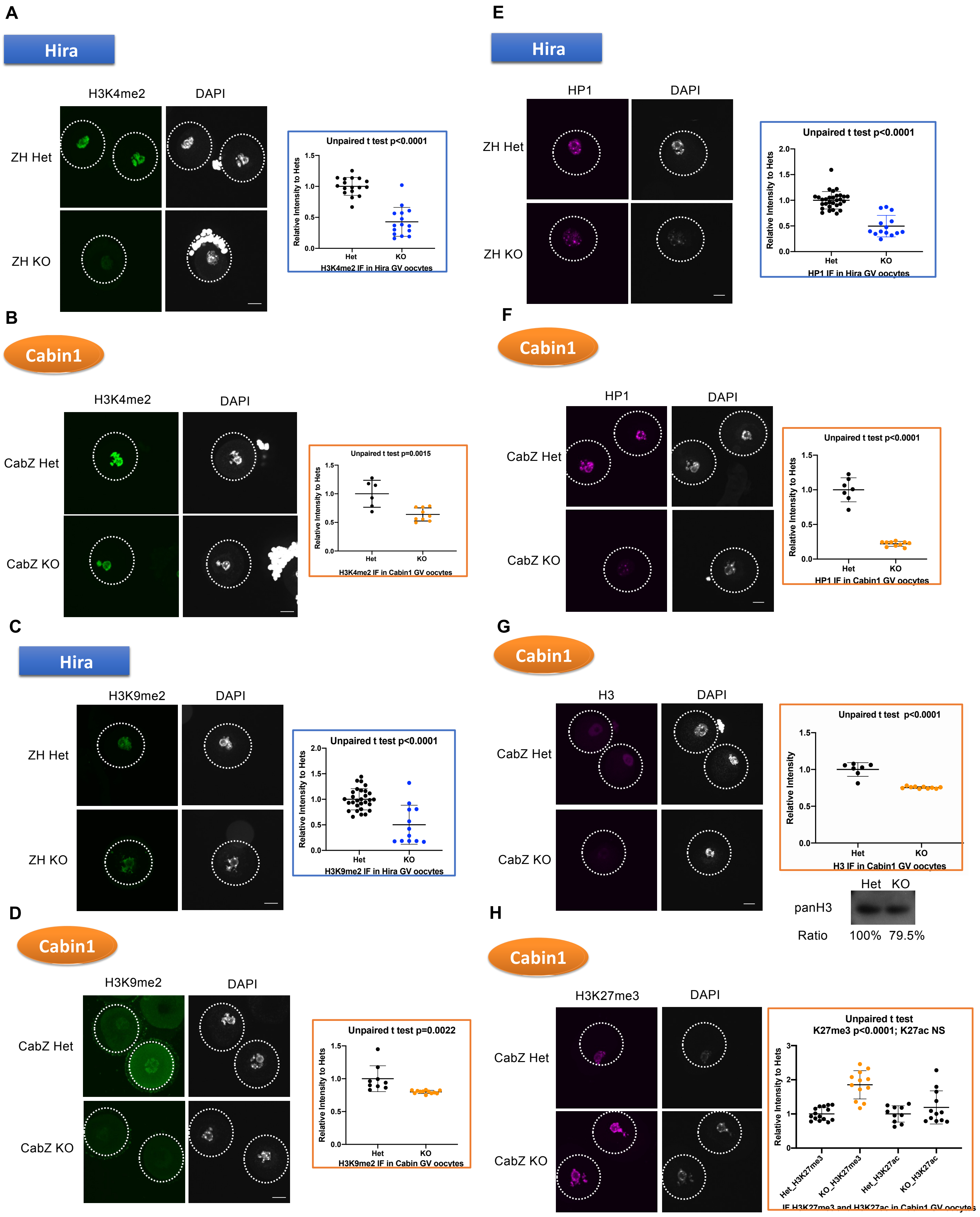

Development • Supplementary information

Fig. S4. Hira complex is required for establishment of repressive histone and heterochromatin marks in GV oocytes.

(A) Hira mutant oocytes fail to establish H3K4me2. Immunofluorescent images (left panel) and quantification (right panel) of H3K4me2 in Hira mutant (ZH KO) and heterozygous control (ZH Het) oocytes.
(B) Cabin1 mutant oocytes fail to establish H3K4me2. Immunofluorescent images (left panel) and quantification (right panel) of H3K4me2 in Cabin1 mutant (CabZ KO) and heterozygous control (CabZ Het) oocytes.
(C) Hira mutant GV oocytes fail to establish H3K9me2. Immunofluorescent images (left panel) and quantification (right panel) of H3K9me2 in ZH KO and ZH Het oocytes.
(D) Cabin1 mutant oocytes fail to establish H3K9me2. Immunofluorescent images (left panel) and quantification (right panel) of H3K9me2 in CabZ KO and CabZ Het oocytes.
(E) Hira mutant oocytes failed to establish HP1. Immunofluorescent images (left panel) and quantification (right panel) of HP1 in ZH KO and ZH Het oocytes.
(F) Cabin1 mutant GV oocytes failed to establish HP1. Immunofluorescent images (left panel) and quantification (right panel) of HP1 in CabZ KO and CabZ Het oocytes. (G) Overall histone H3 level was decreased in Cabin1 mutant oocytes. Immunofluorescent images (left panel), quantification (upper right panel), and western blot (lower right panel) of H3K4me3 in CabZ KO and CabZ Het oocytes.
(H) The level of the H3K27me3 mark was upregulated in Cabin1 mutant GV oocytes while the H3K27ac mark remained. Immunofluorescent images (left panel) of H3K27me3 and quantification (right panel) of H3K27me3 and H3K27ac in CabZ KO and CabZ Het oocytes. Scale bar: 25mm

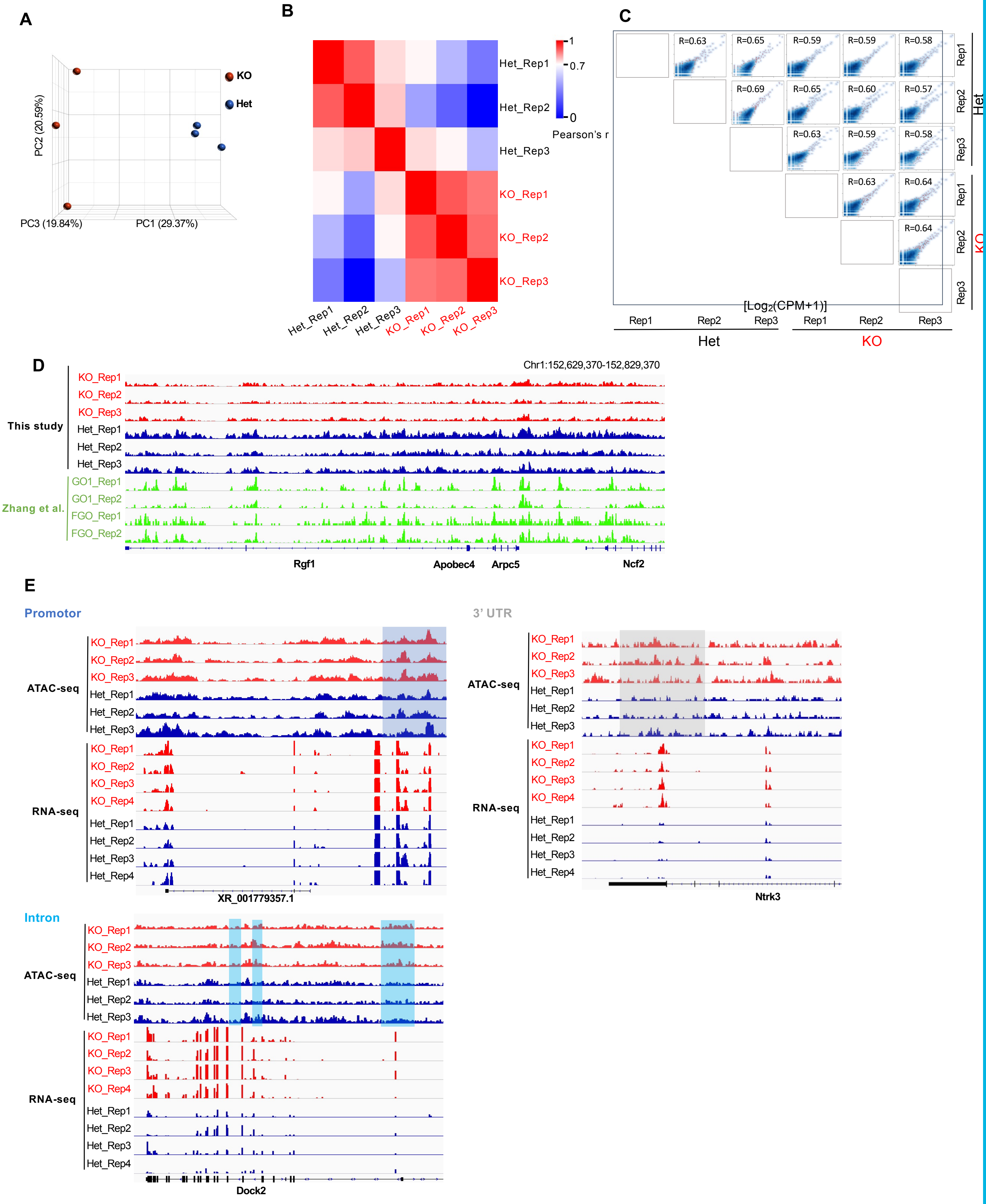
A
KO
Het
PC2 (20.59%)
PC3 (19.84%)
PC1 (29.37%)
B
Het_Rep1
Het_Rep2
Het_Rep3
KO_Rep1
KO_Rep2
KO_Rep3
1
0.7
0
Pearson's r
C
R=0.63
R=0.65
R=0.59
R=0.59
R=0.58
R=0.69
R=0.65
R=0.60
R=0.57
R=0.63
R=0.59
R=0.58
R=0.63
R=0.64
R=0.64
[Log2(CPM+1)]
Rep1
Rep2
Rep3
Het
KO
D
Chr1:152,629,370-152,829,370
This study
Zhang et al.
GO1_Rep1
GO1_Rep2
FGO_Rep1
FGO_Rep2
Rgf1
Apobec4
Arpc5
Ncf2
E
Promotor
3' UTR
ATAC-seq
RNA-seq
KO_Rep4
Het_Rep4
XR_001779357.1
Ntrk3
Intron
Dock2

Fig. S5. Global chromatin accessibility is affected in Cabin1 mutant GV oocytes.

(A) Principal Component Analysis (PCA) of ATAC-seq peaks of Cabin1 oocytes, where the distance among samples was measured by the pairwise Pearson correlation and evaluated using the accessibility of 2,000bp genome bins.

(B) Hierarchical clustering of all samples, where the distance among samples was measured by the Pearson correlation. The colour spectrum, ranging from blue to red, indicates correlation from low to high.
(C) Scatter plots showing the correlation between the three ATAC-seq replicates of CabZ Het and KO oocytes.
(D) The genome browser view showing the enrichment of ATAC-seq peaks in both CabZ Het and KO oocytes, as well as the data from GO1 and FGO oocytes (Zhang et al., 2020) on the locus of chromosome1 152,629,370-152,829,370.
(E) Genome browser showing the ATAC-seq peaks and RNA-seq reads enrichment near gene promoter (i.e. XR_001779357.1), 3'UTR (i.e. *Ntrk3*), and intron (i.e. Dock2) regions.

Development • Supplementary information

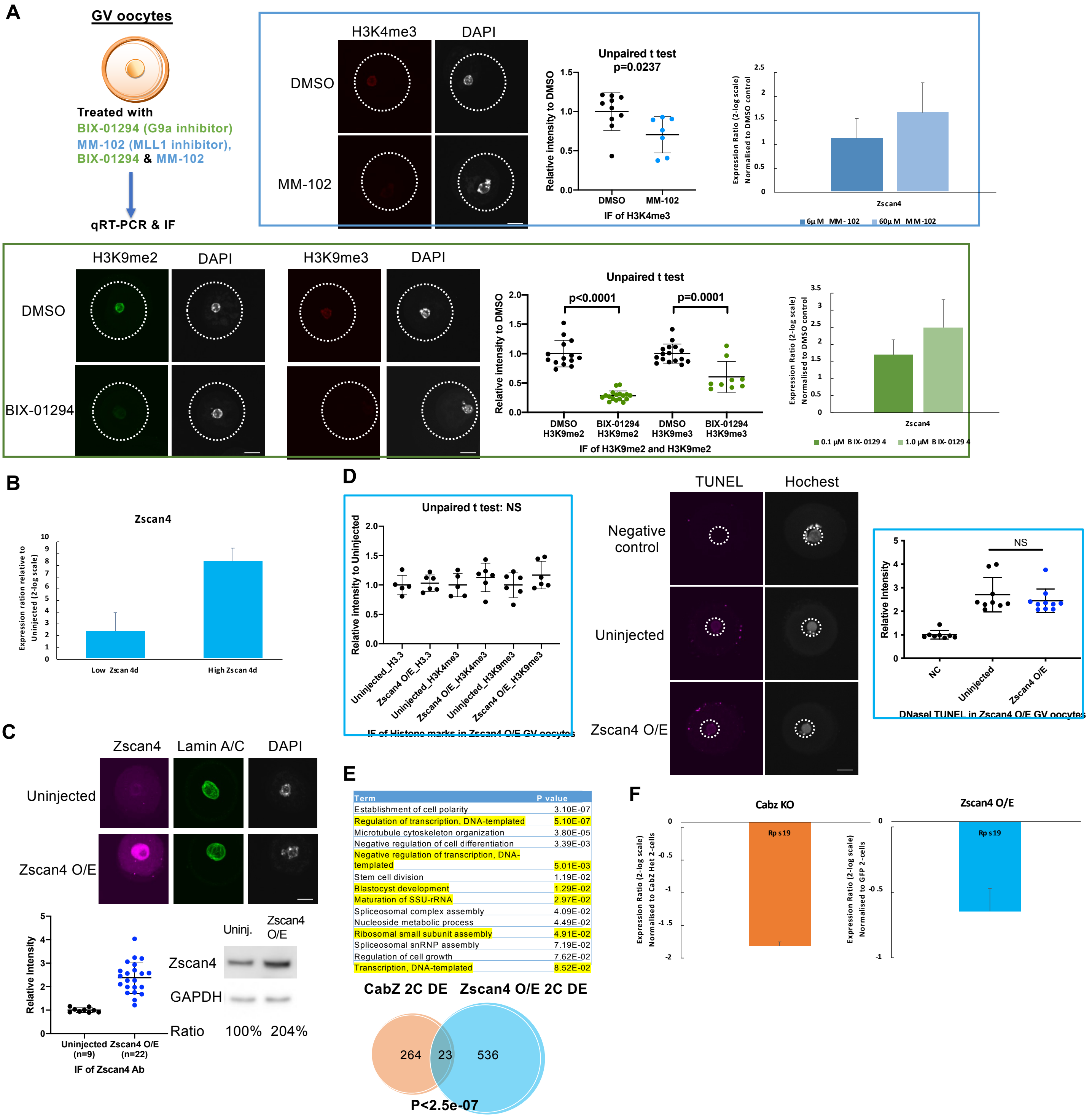

Term	P value
Establishment of cell polarity	3.10E-07
Regulation of transcription, DNA-templated	5.10E-07
Microtubule cytoskeleton organization	3.80E-05
Negative regulation of cell differentiation	3.39E-03
Negative regulation of transcription, DNA-templated	5.01E-03
Stem cell division	1.19E-02
Blastocyst development	1.29E-02
Maturation of SSU-rRNA	2.97E-02
Spliceosomal complex assembly	4.09E-02
Nucleoside metabolic process	4.49E-02
Ribosomal small subunit assembly	4.91E-02
Spliceosomal snRNP assembly	7.19E-02
Regulation of cell growth	7.62E-02
Transcription, DNA-templated	8.52E-02

Development • Supplementary information

Fig. S6. Zscan4 expression is regulated by repressive histone marks of H3K4me3 and H3K9me3.
(A) Histone methyltransferase inhibitor experiments confirmed that Zscan4 expression is modulated by H3K4me3, H3K9me2, and H3K9me3 modifications. Oocytes were treated with BIX-01294 or MM-102 for 48hrs. IF was used to confirm the inhibition of target histone marks. qRT-PCR was used to confirm the upregulation of Zscan4 after inhibitor treatment.
(B) qRT-PCR validation of Zscan4 overexpression in oocytes. RNA concentration of low Zscan4 (300ng/ml) and high (1,500ng/ml).
(C) Confirmation of Zscan4 overexpression. Upper panel: immunofluorescence using antibody against Zscan4. Lower left panel: quantification of the IF images. Lower right panel: Western blot of Zscan4 overexpression in oocytes, GAPDH was used as a loading control (n=2).
(D) Overexpression of Zscan4 was unable to alter the level of H3.3, H3K4me3 and H3K9me3 by IF or global chromatin accessibility by DNaseI-TUNEL assay.
(E) RNA-seq result of overexpressed Zscan4 oocytes at 2-cell stages. Upper panel: Gene Ontology of RNA-seq result of overexpressed Zscan4 (Zscan4 O/E) 2-cells (2C) showed that regulation of transcription, blastocyst development, and ribosomal assembly related terms are among the top downregulated lists. Panel below: Venn-diagram represented the co-downregulated differentially expressed genes of Cabin1 mutant and overexpressed Zscan4 oocytes at 2-cell stages. For comparison, differentitial expression cutoff threshold has a false discovery rate (FDR) adjusted p-value of <0.05 and fold change >2. Significance of overlap assessed by hypergeometric test.
(F) qRT-PCR of CabZ KO and Zscan4 overexpressed 2-cells confirmed the downregulation of Rps19. Scale bar= 25mm.

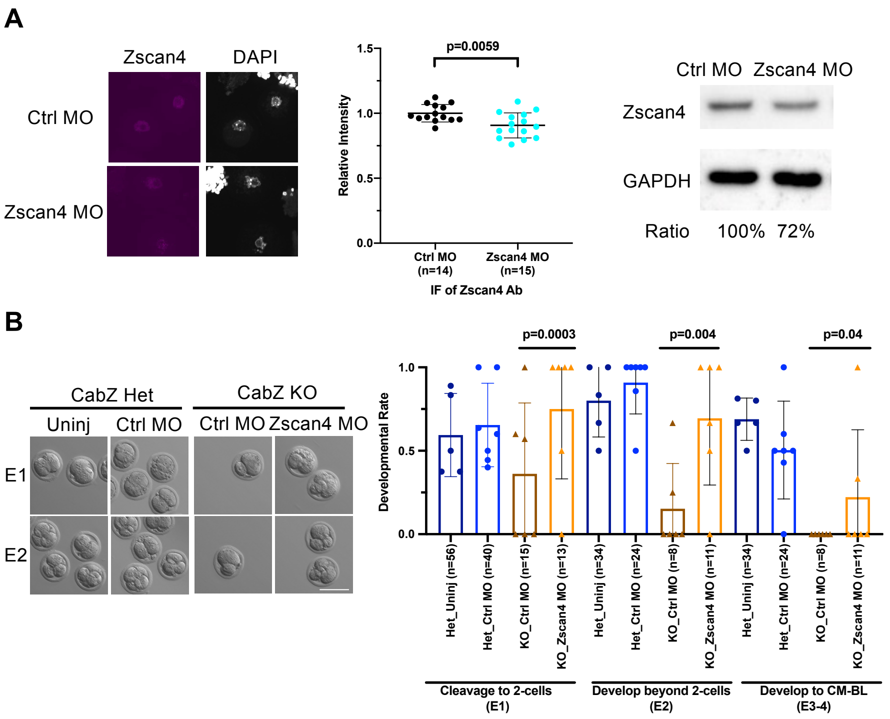

Fig. S7. Downregulation of Zscan4 partially rescues the developmental potential of Cabin1 mutant oocytes.

(A) Validation of Zscan4 knockdown in wild-type oocytes. Left panel: IF images and quantification of Zscan4 in Control morpholino (Ctrl MO) and Zscan4 MO injected oocytes. Right panel: Western blot and quantification of Zscan4 in Ctrl MO and Zscan4 MO injected oocytes. GAPDH was used as a loading control (n=2).

(B) Developmental progression of parthenogenesis embryos of CabZ Het and KO oocytes following morpholino microinjection. Left panel: Representative images of CabZ Het and KO oocytes under different treatments on embryonic day1 (E1) and day2 (E2).

Right panel: Quantification of developmental progression of embryos following microinjections. CabZ KO GV oocytes were microinjected with Ctrl and Zscan4 MO; CabZ Het GV oocytes were uninjected (Uninj) or injected with Ctrl MO, respectively. After injection, the oocytes were matured to MII followed by parthenogenesis, then monitored for cleavage and subsequential embryonic development. Developmental progressions were monitored on E1 by 2-cell formation, on E2 by development beyond 2-cells and on E3-E4 by development to morula-to-blastocyst stages. χ^2 p values were calculated for the developmental rate of Cabin1 KO embryos injected with Zscan4 and Control morpholinos. Scale bar=80mm.

Development • Supplementary information

Table S1. Summary of ATAC-seq library mapping and data processing.

Sample ID	No. of paired-end reads	No. of mapped reads	Mapping rates (%)	No. of ATAC-seq peaks
CabZ Het_1	61728101	59691073	96.7	43399
CabZ Het_2	43749714	42502847	97.15	67074
CabZ Het_3	53986271	52830964	97.86	111327
CabZ KO_1	46356933	43524524	93.89	46070
CabZ KO_2	52255421	50483962	96.61	19160
CabZ KO_3	63228286	60907807	96.33	29600

Table S2. Gene ontology analysis of the Cabin1 GV oocytes ATAC-seq data.

Click here to download Table S2

Table S3. Integrated analysis of the ATAC-seq and the RNA-seq datasets.

Click here to download Table S3

Table S4. List of antibodies used in this study.

	Manufacturer	Cat. No	Dilution
CABIN1	Abcam	3349	1:500 for WB
CREST	Immunivision	HCT-0100	1:500
GAPDH	Santa Cruz	97166	1:500
H3	Abcam	ab1791	1:250 for IF; 1:500 for WB
H3K27ac	Abcam	ab177178	1:1000
H3K27me3	Diagenode	C15410195	1:200
H3K4me2	Cosmo Bio	MCA-MABI0003	1:1000
H3K4me3	Diagenode	1541003	1:500 for WB
H3K4me3	Millipore	04-745	1:250
H3K9me2	Abcam	ab1220	1:100
H3K9me3	Abcam	ab8898	1:500
HP1	Cell Signaling	8676T	1:250
Laimn A/C	Sigma	SAB 4200236	1:500
PolII PS2	Abcam	193468	1:100
Zscan4	Gift	Minoru Ko lab	1:250 for IF
Zscan4	Abcam	97748	1:250 for WB
Peroxidase Donkey Anti-Rabbit	Jackson Immunoresearch	#711-035-152	1:10,000
Alexa Fluor 488 Donkey anti mouse	ThermoFisher	A21202	1:500
Alexa Fluor 568 Donkey anti rabbit	ThermoFisher	A10042	1:500
Alexa Fluor 647 Donkey anti mouse	Abcam	ab150107	1:500

Development • Supplementary information

Table S5. List of primers used in this study.

Zscan4 (d)	Forward	TCCGTAGAGATGCCAAACTATTC
	Reverse	GACAGGTGACACAAAGCAATTC
Zscan4 (C)	Forward	TTGAAGCCTCCTGTCATGGTCC
	Reverse	TCCATTTCATTTCCACTACAGC
Ddit3	Forward	TGTTGAAGATGAGCGGGTG
	Reverse	AGGTTCTGCTTTCAGGTGTG
Gm5039	Forward	CCACTGTTAGATACTTCCTGGC
	Reverse	AAAAGGTAACCACAGGATCCG
Rps19	Forward	AAGTCCGGGAAGCTGAAAG
	Reverse	GAAGCAGCTCGTGTGTAGAA
