# Supplementary material for: The H3.3 chaperone Hira complex orchestrates oocyte developmental competence
Source: Development. 2022 Feb 28;149(5):dev200044. doi: 10.1242/dev.200044 (PMC8959146; doi:10.1242/dev.200044)
Supplement: Reviewer comments [file develop-149-200044_review_history.pdf]

The H3.3 chaperone Hira complex orchestrates oocyte developmental competence

Rowena Smith, Andrej Susor, Hao Ming, Janet Tait, Marco Conti, Zongliang Jiang and Chih-Jen Lin
DOI: 10.1242/dev.200044

Editor: Haruhiko Koseki

Review timeline

Original submission:	21 July 2021
Editorial decision:	14 September 2021
First revision received:	10 December 2021
Editorial decision:	7 January 2022
Second revision received:	7 January 2022
Accepted:	16 January 2022

Original submission

First decision letter

MS ID#: DEVELOP/2021/200044

MS TITLE: The H3.3 chaperone Hira complex orchestrates oocyte developmental competence

AUTHORS: Rowena SSmith, Andrej Susor, Hao Ming, Janet Tait, Marco Conti, Zongliang Jiang, and Chih-Jen Lin

I have now received all the referees' reports on the above manuscript, and have reached a decision. The referees' comments are appended below, or you can access them online: please go to BenchPress and click on the 'Manuscripts with Decisions' queue in the Author Area.

As you will see, the referees express considerable interest in your work, but have some significant criticisms and recommend a substantial revision of your manuscript before we can consider publication. If you are able to revise the manuscript along the lines suggested, which may involve further experiments, I will be happy receive a revised version of the manuscript. Your revised paper will be re-reviewed by one or more of the original referees, and acceptance of your manuscript will depend on your addressing satisfactorily the reviewers' major concerns. Please also note that Development will normally permit only one round of major revision.

We are aware that you may be experiencing disruption to the normal running of your lab that make experimental revisions challenging. If it would be helpful, we encourage you to contact us to discuss your revision in greater detail. Please send us a point-by-point response indicating where you are able to address concerns raised (either experimentally or by changes to the text) and where you will not be able to do so within the normal timeframe of a revision. We will then provide further guidance. Please also note that we are happy to extend revision timeframes as necessary.

Please attend to all of the reviewers' comments and ensure that you clearly highlight all changes made in the revised manuscript. Please avoid using 'Tracked changes' in Word files as these are lost in PDF conversion. I should be grateful if you would also provide a point-by-point response detailing how you have dealt with the points raised by the reviewers in the 'Response to Reviewers' box. If you do not agree with any of their criticisms or suggestions please explain clearly why this is so.

© 2022. Published by The Company of Biologists under the terms of the Creative Commons Attribution License (https://creativecommons.org/licenses/by/4.0/).

Reviewer 1

Advance summary and potential significance to field

In this manuscript, the authors investigate the role of maternal Hira complex in oocyte developmental competence. They found that knockout or knockdown of any subunit of the Hira complex during mouse oocyte development leads to early embryogenesis failure. Mechanistically, embryos without the Hira complex fail to silence global transcription, due to compromised H3.3 deposition, reduced repressive H3K4me3 and H3K9me3 histone marks, and aberrant chromatin accessibility. Moreover, the authors demonstrated that the elevated expression of Zscan4 in these mutant embryos is responsible for the embryogenesis failure. Overall, the role and mechanism of the Hira complex in regulating oocyte developmental competence is of significance for the field of developmental biology. However, some conclusion is not fully supported by the current data.

Comments for the author

Major concern:
The conclusion that the elevated expression of Zscan4 in the Hira mutant embryos is responsible for the embryogenesis failure, is not solid. First, the authors need to show that the Zscan4 expression level in the Zscan4 overexpression embryos is similar to the elevated Zscan4 levels in the ZH KO, CabZ KO, and Ubn1 MO embryos. Secondly, it is necessary to demonstrate that knockdown of Zscan4 in the ZH KO, CabZ KO, or Ubn1 MO embryos allows normal development.

Minor comments:
1. Some data is missing or not correctly cited. For example, line 405-406, “very little changes of key histone marks (H3.3, H3k4me3, and H3K9me3) was observed by IF in the Zscan4 overexpressed oocytes (Fig.6D)”.
2. In some cases, the connection between sentences is not logically flow. For example, line 207-210, “Notably, among top-ranked GO terms from up-regulated genes was an enrichment of a set of telomere related genes (Fig. 2C). We further compared our data with 2-cell specific gene datasets generated by Wu et al (Wu et al., 2016)”. What is the connection between telomere related genes and 2-cell specific genes?
3. Some obvious typos and grammar errors. Line 199 “wereidentical” and line 200 “bothe”. Line 174-175, “A greater percentage of the 57.5% of upregulated genes in CabZ mutants overlap with upregulated genes in ZH mutants”.

Reviewer 2

Advance summary and potential significance to field

In this paper, the authors showed that KO of the Hira complex results in gene derepression in fully grown oocytes (FGOs), accompanied by reduction of various histone modifications and changes in chromatin accessibility. The derepressed genes included ZGA-marker genes such as Zscan4. Inhibitor-mediated reduction of H3K4me3 and H3K9me3 could induce derepression of Zscan4, supporting the notion that the gene derepression in the mutants may be caused by defective establishment of repressive histone modifications in oocytes. Zscan4 overexpression partly recapitulated the phenotypes of CabZ KO mutants including reduction of protein synthesis, transcriptome changes, and developmental arrest. Together, this study suggests that the Hira complex is required for proper gene silencing in FGOs, and thus required for developmental competence.

I found this to be potentially interesting to the reproduction and chromatin field. Meanwhile, there are several concerns.

Comments for the author

1. ATAC-seq data Based on the bigwig files deposited by the authors, the signal/noise ratio is quite poor when compared to previously published ATAC-seq data of GV oocytes from the other groups. Therefore, the data quality should be more thoroughly validated (not just by PCA analysis). For

© 2022. Published by The Company of Biologists under the terms of the Creative Commons Attribution License (https://creativecommons.org/licenses/by/4.0/).

example, scatterplots of ATAC peak signals compared (1) between each replicate and (2) between the current data and some of published data are necessary. Moreover, the representative tracks of Het and KO data shown in Fig 5E and S5C appear to be intentionally picked: There are 3 replicates in both Het and KO, and the replicates are poorly correlated at these loci (except Rbm15b). It appears that the authors intentionally picked each of the replicates that are in line with the story, while ignoring the fact that the other replicates are inconsistent. Therefore, these figures should be removed.

2. Abstract Several statements are a bit too strong despite the lack of evidence. First, ‘mutant oocytes fail to silence global transcription’ should be ‘transcription is not fully silenced in mutant oocytes’. Second, ‘Hira complex mutants are unable to establish the H3K4me3 and H3K9me3, resulting in aberrant chromatin accessibility’
should be ‘H3K4me3 and H3K9me3 are reduced and accessible chromatin landscape is affected in Hira complex mutants’. Importantly, the original statement implicates the order of mechanistic action –defective histone modifications cause aberrant chromatin accessibility-, however, this study did not reveal this order but rather only show cooccurrence. The inhibitor experiment does not support this order, as it only showed Zscan4 derepression but not aberrant chromatin accessibility. Lastly, ‘Overexpression of Zscan4 in the oocytes recapitulates the phenotypes of Hira mutants’ should be ‘Overexpression of Zscan4 partially recapitulates the phenotypes of Hira mutants’.

3. line152

>Thus, the Hira complex is responsible for the acquisition of developmental competence in mouse oocytes the corresponding genes functioning as maternal-effect genes.

I couldn’t understand what does ‘the corresponding genes functioning as maternal-effect genes’ mean.

Better to remove. Also, “responsible” is not appropriate, as Hira complex is one of many requirements for developmental competence. I recommend the term “required” instead of “responsible”.

4. line 222

>We chose to focus on Zscan4 as a candidate gene since it is involved in telomere maintenance.

“Candidate” for what means? Does this mean that Zscan4 is a candidate derepressed gene that might account for developmental failure?

5. Fig 3A/B.

Zscan4 is upregulated in ZH KO and CabZ KO oocytes. Since Zscan4 is known to be regulated by DUX, does the authors check if DUX is also upregulated? Regardless of the result, this information deserves to be included.

6. Fig 3C/D. Is the quantification data measuring the signals only in SN oocytes? If so, state it in figure legends. If not, quantification of only SN oocytes is necessary because SN and NSN are very different in transcriptional activity.

7. line 269

>Recent research indicates that two main histone repressive marks in this context, H3K9me3 and H3K4me3 (Dahl et al., 2016), (Zhang et al., 2016), are responsible for the transcriptional quiescence in SN oocytes (Dumdie et al., 2018a).

I am not aware that these papers demonstrate the responsibility of H3K9me3 in transcriptional quiescence in oocytes (H3K4me3 was demonstrated). So, need to rephase this part.

8. Fig S6.

Data of the inhibitor-mediated derepression of Zscan4 is one of the main results in this story. So, I recommend to move the Fig S6A-red (mixed inhibitor experiment) to a main figure. Additionally, since the bar graphs which lack bars of the control groups are not easy to understand, the bar of control groups should be added.

9. line 405 Fig 6B, not Fig 5B.

10. line 425 Fig 6E, not 6C

© 2022. Published by The Company of Biologists under the terms of the Creative Commons Attribution License (https://creativecommons.org/licenses/by/4.0/).

First revision

Author response to reviewers' comments

Point-to-Point Response

We would like to thank both Reviewers for their valuable comments (in **bold black colour**), which have helped us improve the manuscript significantly. We have addressed the points (in blue colour) raised by each of the Reviewers with new data, as described below.

Reviewer 1 Advance Summary and Potential Significance to Field:

In this manuscript, the authors investigate the role of maternal Hira complex in oocyte developmental competence. They found that knockout or knockdown of any subunit of the Hira complex during mouse oocyte development leads to early embryogenesis failure. Mechanistically, embryos without the Hira complex fail to silence global transcription, due to compromised H3.3 deposition, reduced repressive H3K4me3 and H3K9me3 histone marks, and aberrant chromatin accessibility. Moreover, the authors demonstrated that the elevated expression of Zscan4 in these mutant embryos is responsible for the embryogenesis failure. Overall, the role and mechanism of the Hira complex in regulating oocyte developmental competence is of significance for the field of developmental biology.

However, some conclusion is not fully supported by the current data.

We would like to thank Reviewer 1 for the positive comments, and we have provided new data and addressed the comments raised by the reviewer (see below).

Reviewer 1 Comments for the Author:

Major concern:

The conclusion that the elevated expression of Zscan4 in the Hira mutant embryos is responsible for the embryogenesis failure, is not solid. First, the authors need to show that the Zscan4 expression level in the Zscan4 overexpression embryos is similar to the elevated Zscan4 levels in the ZH KO, CabZ KO, and Ubn1 MO embryos.

Thanks for the comment and we apologise for not providing the details.

Firstly, we have validated the level of Zscan4 in Zscan4 over-expression oocytes, ZH KO, CabZ KO, and Ubn1KD GV oocytes using qRT-PCR and protein assays (western blot and/or IF). The results of qRT-PCR show Zscan4 RNA upregulated by: Log 2.32 fold change (FC) for Zscan4 overexpression oocytes, Log 2.02 for CabZ KO, and Log 2.13 for ZH KO (Fig S2B and **new Fig. S6B**). The increase in protein levels of Zscan4 in Zscan4 overexpression oocytes is between ~200% (by western blot) and ~230% (by IF; Fig S6C); the change for ZH KO is ~142% (by IF in Fig 3A) and the change for CabZ KO between ~151% (by IF) and ~170% (by western-blot in Fig 3B). For Ubn1, because we injected Ubn1 MO to fully-grown GV stage oocytes, the inhibition effect might not be as strong as the genetic approach which depletes Cabin1 and Hira during oogenesis. This could therefore result in a milder upregulation level of Zscan4 (RNA level: Log2 FC for Ubn1 KD: 1.23; Fig S2B). We have added the values into the main text in **line 369-374**. In brief, we believe the Zscan4 overexpression approach results in an acceptable range which is similar to the level observed in mutants in the oocytes.

Whether this abnormal upregulation phenomenon will persist from oocytes to embryos is an outstanding question. As Reviewer1 suggested, for evaluation of the level of Zscan4, we have performed qRT-PCR of Cabin1 Het 2PN and Cabin1 KO 1PN zygotes and Hira Het 2PN and Hira KO 1PN zygotes to compare with Uninjected and Zscan4 overexpressed embryos (generated by parthenogenesis) and then used wild-type zygotes and 2-cells as controls.

As we can see from the result (**new Fig. S3C**), we detected the upregulation of Zscan4 in both CabZ KO 1PN compared to CabZ Het 2PN zygotes (Log 1.225=2.38 fold) and ZH KO 1PN compared to ZH Het 2PN zygotes (Log 1=2 fold). We did observe a higher upregulation of Zscan4 in the Zscan4 over-

© 2022. Published by The Company of Biologists under the terms of the Creative Commons Attribution License (https://creativecommons.org/licenses/by/4.0/).

expression embryos, we think this is due to the RNA stabilisation modifications from the in vitro transcription process (Capping and polyadenylation steps) which led to higher protein yields. However, the new result showed the persistent elevated Zscan4 level in the mutant embryos and supports the notion that elevated expression of Zscan4 led to the embryogenesis failure.

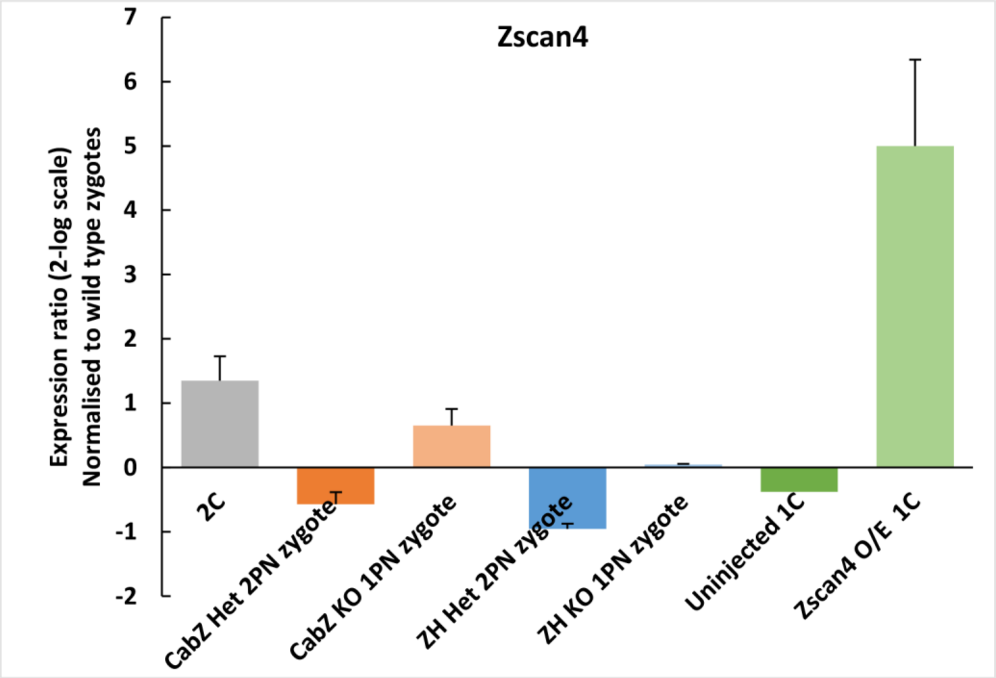

Secondly, it is necessary to demonstrate that knockdown of Zscan4 in the ZH KO, CabZ KO, or Ubn1 MO embryos allows normal development.

Regarding knockdown of Zscan4 in the loss-of-function Hira complex oocytes (e.g. Cabin1 KO) rescue experiment, we agree with the Reviewer that it would be an important experiment. However, there are significant technical difficulties associated with it.

Firstly, the highly repetitive sequences of multiple copies of Zscan4 genes (Zscan4a, Zscan4b, Zscan4c, Zscan4d, Zscan4e, and Zscan4f) and pseudogenes (Zscan4-ps1, Zscan4-ps2, and Zscan4-ps3), in the mouse Zscan4 genomic cluster, have created an obstacle for genetic study (traditional knockout or CRISPR) of the Zscan4 genes.

NOTE: We have removed unpublished data that had been provided for the referees in confidence.

Secondly, it is widely accepted that it is difficult to achieve this by RNAi introduction into GV- or MII-stage oocytes because of the high stability of target proteins. An alternative approach, of collecting and injecting siRNA into follicles from 12-day-old mice, then performing in vitro growth, in vitro maturation, and fertilisation, and monitoring the preimplantation development has been used (Inoue and Zhang, 2014 Nat Stru Mol Biol). However, complicated manipulations (microinjection, in vitro follicle growth, in vitro maturation, in vitro fertilisation/parthenogenesis, in vitro culture) are involved. More importantly, the final developmental outcome is very poor. There was only ~20-40% blastocyst formation rate in a B6D2 F1 hybrid strain. We expect that it would be an even lower developmental rate if applied to the C57B/6 inbred strain that we use. Therefore, it is not appropriate to apply this methodology to our experiment (i.e., knockdown Zscan4 by siRNA in the Cabin1 Het and KO growing oocytes within follicles, perform IVM, parthenogenesis, and then monitor the in vitro developmental outcome).

© 2022. Published by The Company of Biologists under the terms of the Creative Commons Attribution License (https://creativecommons.org/licenses/by/4.0/).

Another approach is our established morpholino antisense oligo knockdown platform (Chen et al., 2013, NCB; Lin et al., 2013 Dev; 2014 Dev Cell etc.) to introduce Zscan4 morpholino into the GV oocytes and thus block Zscan4 mRNA translation.

Prior to performing the Zscan4 knockdown experiment, we mined two independent datasets generated from our team, each using a completely different approach (polysome fractionation RNA-seq dataset from the Susor Group and a new method for quantification of translation efficiency by using RiboTaq and RNA-seq approach developed by the Conti Group). Both indicated that the translation efficiency of Zscan4 is lower compared to other maternal effect genes, echoing our observation that Zscan4 is repressed. Therefore, we believe using morpholino knockdown to deplete Zscan4 in oocytes is the optimum method available. With this caveat in mind, indeed, we could only achieve Zscan4 knockdown of ~30% (quantification by IF and Western blot) in the wild-type samples **new Fig. S7A.**

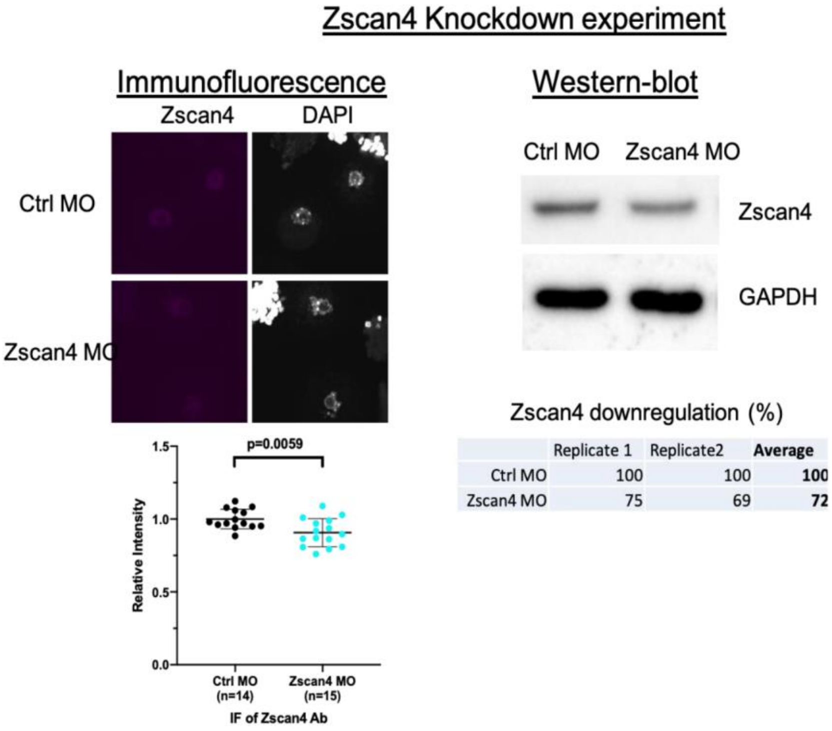

	Replicate 1	Replicate2	Average
Ctrl MO	100	100	**100**
Zscan4 MO	75	69	**72**

Figure Validation of Zscan4 knockdown in wild-type oocytes. Left panel: IF images and quantification of Zscan4 in control MO and Zscan4 MO injected oocytes.
Right panel: Western blot and quantification of Zscan4 in control MO and Zscan4 MO injected oocytes.

Albeit challenging, we used this approach to perform rescue experiments by microinjecting Control and Zscan4 morpholinos (MO) into CabZ KO GV oocytes (comparing them to Uninjected and Ctrl MO injected CabZ Het GV oocytes). After injection, the oocytes were matured to MII followed by parthenogenesis, then monitored for cleavage and subsequential embryonic development.

Our result shows that CabZ Het GV oocytes (both Uninjected and those which underwent control MO injection) can successfully cleave, develop beyond 2-cells and develop to compacted morula and blastocyst stages. By contrast, CabZ KO GV oocytes (injected with Control MO) reveal impaired developmental potential by showing a very low propensity of embryos reaching 2-cell, and/or developing beyond 2-cells and with resulting failure to form more advanced pre-implantation stages. Importantly, after microinjection of Zscan4 MO into CabZ KO oocytes, we could partially but significantly restore the development of embryos by improving the cleavage rate, development beyond 2-cells and progress to more advanced stages.
CabZ KO oocytes are developmentally incompetent, as demonstrated by their lack of developmental potential and by the RNA-seq dataset. We also noted that, after microinjection, they are more fragile than CabZ Het oocytes. Therefore, far fewer oocytes manage to survive

© 2022. Published by The Company of Biologists under the terms of the Creative Commons Attribution License (https://creativecommons.org/licenses/by/4.0/).

microinjection, in vitro maturation, and parthenogenesis. In addition, because our animal colonies were downsized due to the COVID crisis, we did not have enough mice to obtain high numbers of oocytes/embryos.

We considered that insufficient knockdown would lead to a partial rescue of developmental outcome which potentially did not reflect faithfully the conditions after depletion of Zscan4, and so we decided to include part of the result in **new Fig S7B.**

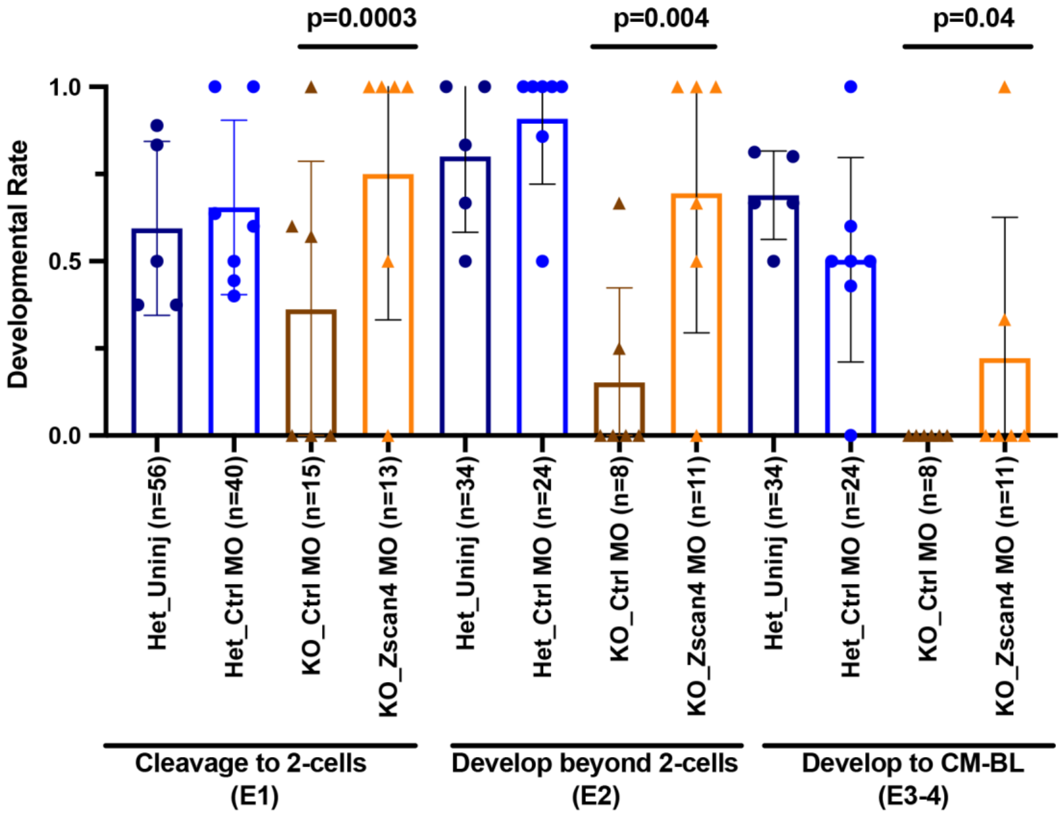

The temporal fine-tuning regulation of Zscan4 is essential for oogenesis (from this study) and zygotic genome activation. We believe a study to pursue the consequences after acute-depletion of Zscan4 at various stages by, for example, using the TrimAway approach is well deserved. However, conventional co-microinjection of Trim21 mRNA with an antibody does not deplete nuclear protein like Zscan4. An additional treatment, by fusion of the Fc-domain of an antibody to a nanobody tag is required. The aforementioned challenges should be pursued but lie beyond the scope of this present study.

With the combination of showing the elevation of Zscan4 in the mutant Cabin1 and Hira embryos mentioned above and the new rescue data of Zscan4 knockdown in the Cabin1 mutant oocytes, we believe that we have provided sufficient evidence to demonstrate that repression of Zscan4 in the oocyte is critical for oocyte-to-embryo transition. We hope the reviewer can appreciate this.

Minor comments:
1. Some data is missing or not correctly cited. For example, line 405-406, "very little changes of key histone marks (H3.3, H3k4me3, and H3K9me3) was observed by IF in the Zscan4 overexpressed oocytes (Fig.6D)".

We apologise for this typographic error. We have corrected it to **new Fig. S6D**.

2. In some cases, the connection between sentences is not logically flow. For example, line 207-210, "Notably, among top-ranked GO terms from up-regulated genes was an enrichment of a set of telomere related genes (Fig. 2C). We further compared our data with 2-cell specific gene datasets generated by Wu et al (Wu et al., 2016)". What is the connection between telomere related genes and 2-cell specific genes?

© 2022. Published by The Company of Biologists under the terms of the Creative Commons Attribution License (https://creativecommons.org/licenses/by/4.0/).

We apologise for the lack of clarity. We have revised the text. See **line 195-198.**

3. Some obvious typos and grammar errors. Line 199 "wereidentical" and line 200 "bothe".

We apologise for these typographic errors. We have corrected them.

Line 174-175, "A greater percentage of the 57.5% of upregulated genes in CabZ mutants overlap with upregulated genes in ZH mutants".

We have revised it. See **line 165-167**.

Reviewer 2 Advance Summary and Potential Significance to Field:

In this paper, the authors showed that KO of the Hira complex results in gene derepression in fully grown oocytes (FGOs), accompanied by reduction of various histone modifications and changes in chromatin accessibility. The derepressed genes included ZGA-marker genes such as Zscan4. Inhibitor-mediated reduction of H3K4me3 and H3K9me3 could induce derepression of Zscan4, supporting the notion that the gene derepression in the mutants may be caused by defective establishment of repressive histone modifications in oocytes. Zscan4 overexpression partly recapitulated the phenotypes of CabZ KO mutants, including reduction of protein synthesis, transcriptome changes, and developmental arrest. Together, this study suggests that the Hira complex is required for proper gene silencing in FGOs, and thus required for developmental competence. I found this to be potentially interesting to the reproduction and chromatin field.

Meanwhile, there are several concerns.

We would like to thank Reviewer 2 for the positive comments, and we have provided new data and addressed the comments raised by the reviewer (see below).

Reviewer 2 Comments for the Author:

1. ATAC-seq data
Based on the bigwig files deposited by the authors, the signal/noise ratio is quite poor when compared to previously published ATAC-seq data of GV oocytes from the other groups. Therefore, the data quality should be more thoroughly validated (not just by PCA analysis). For example, scatterplots of ATAC peak signals compared (1) between each replicate and (2) between the current data and some of published data are necessary. Moreover, the representative tracks of Het and KO data shown in Fig 5E and S5C appear to be intentionally picked: There are 3 replicates in both Het and KO, and the replicates are poorly correlated at these loci (except Rbm15b). It appears that the authors intentionally picked each of the replicates that are in line with the story, while ignoring the fact that the other replicates are inconsistent. Therefore, these figures should be removed.

We thank the reviewer for prompting us to further assess the quality of our ATAC-seq data. We now have included new ATAC-seq analyses as suggested.

(1). In addition to PCA analysis, we performed Hierarchical clustering analysis of all samples (three replicates of both CabZ Het and KO GV oocytes), where the distance among samples was measured by the Pearson correlation. This analysis shows the correlation of replicates are over 0.8 for both CabZ Het and KO (**New Fig. S5B**), suggesting higher reproducibility of the data.

(2). We also performed scatter plots analysis and showed the correlation between the three ATAC-seq replicates of Het and KO GV oocytes. The correlation among replicates of both CabZ Het and KO is ranging from 0.63 to 0.69 (**New Fig. S5C**), which is comparable to the recently published GV oocytes ATAC-seq datasets (In Zhang et al., Genes Dev, 2020 study, their scatter plots analysis showed the correlation from two replicates of WT and KO GV oocytes is 0.785 and 0.697 respectively (also see their Figure S7A)).

© 2022. Published by The Company of Biologists under the terms of the Creative Commons Attribution License (https://creativecommons.org/licenses/by/4.0/).

(3). We also conducted a genome browser view to show the enrichment of ATAC-seq peaks from individual replicates of both CabZ Het and KO oocytes, and compared them to the ATAC-seq data from GV oocytes and fully grown GV oocytes from Zhang et al. 2020 (**New Fig. S5D**). Generally, the replicates are very consistent, and the peaks captured in oocytes from Zhang et al., 2020 were present in our study.

(4). We have included three replicates for both CabZ Het and KO GV oocytes in the analysis. We have identified a large number of ATAC-seq peaks (on average 31,610 for KO, and 73,933 for Het oocytes) (Supple Table 1), which is comparable to the published ATAC-seq dataset in mouse oocytes (Wu et al., Nature, 2016 and Zhang et al., 2020), although we agree with the reviewer that the signal/noise ratio is a little bit higher in our datasets compared to ones from Zhang et al., 2020.

The higher noise and differences when comparing our data to other published ones may be due to starting with a lower number of cells (less than 100 oocytes per replicate) and unexpected disruption during the pandemic. We were forced to reduce the animal colonies due to shortage of staff. In order to collect enough oocytes for each replicate, we needed to prolong the sample storage time in the freezer which might result in reduction of the integrity of the sample and increase the batch-to-batch variations. I hope the reviewer appreciates that continuous challenges remain for ATAC-seq analysis of scarce materials such as oocytes.

We also thank the reviewer for constructive comments on the representative tracks. In our original submission, we combined peaks from three replicates for the genome browser view. We acknowledge that variations are present in our replicates for individual loci, we have now removed all genome browser view results (original Figure 5E and Figure S5B as reviewer suggested) and presented individual replicates of representative loci **(New Fig. S5E**).

Together, we have provided additional analysis to validate the quality of our ATAC-seq data. And we have confirmed the genome-wide alteration of the chromatin accessibility, as well as the differential chromatin occupancy at loci linked to transcription and embryo development. The new data consistently correlate with RNA-seq gene expression data and further strengthen the role of Cabin1 in modulating chromatin landscape.

2. Abstract
Several statements are a bit too strong despite the lack of evidence.

First, 'mutant oocytes fail to silence global transcription' should be 'transcription is not fully silenced in mutant oocytes'.

We have corrected it accordingly.

Second, 'Hira complex
mutants are unable to establish the H3K4me3 and H3K9me3, resulting in aberrant chromatin accessibility' should be 'H3K4me3 and H3K9me3 are reduced and accessible chromatin landscape is affected in Hira complex mutants'.

We have corrected it accordingly.

Importantly, the original statement implicates the order of mechanistic action -defective histone modifications cause aberrant chromatin accessibility-, however, this study did not reveal this order but rather only show cooccurrence. The inhibitor experiment does not support this order, as it only showed Zscan4 derepression but not aberrant chromatin accessibility.

We thank the reviewer for the constructive comment and apologise for the confusion.
In the original manuscript version: **line 413**: Depletion of Cabin1 causes the reduction of repressive histone marks (i.e. H3K4me3 and H3K9me3) **coupled with** abnormal openness of accessible chromatin loci that leads to misregulation of maternal transcripts (e.g. Zscan4).

© 2022. Published by The Company of Biologists under the terms of the Creative Commons Attribution License (https://creativecommons.org/licenses/by/4.0/).

To further demonstrate the mechanism of action we have added new experimental data.

1. Firstly, we examined whether the global chromatin accessibility would be remodelled triggered by Zscan4 upregulation. To do this, we overexpressed Zscan4 in GV oocytes and examined the overall chromatin accessibility using a DNaseI-TUNEL assay which showed no significant change in the level of global chromatin accessibility. This data together with the unchanged histone marks upon Zscan4 overexpression (in **New Fig. S6D**) supports that Zscan4 is downstream of the cascades of both histone modifications and chromatin landscape alteration.

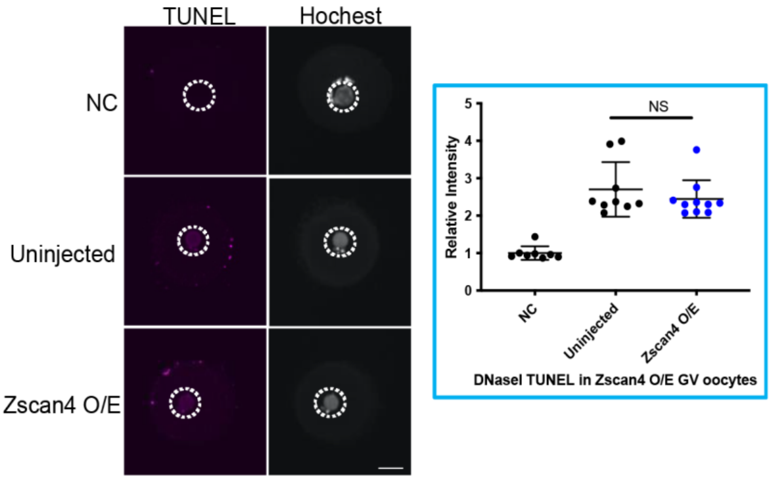

Figure Overexpression of Zscan4 was unable to alter global chromatin accessibility.

2. Secondly, we added a drug inhibition experiment (BIX-01294+MM-102) on wild-type GV oocytes to test whether the global chromatin landscape would change. Very interestingly, we observed that the global chromatin accessibility increased upon treatment which suggests the repressive H3K4me3 and H3K9me3 histone marks maintain the global chromatin integrity. Forced removal of these marks result in alteration of the chromatin decondensation status.

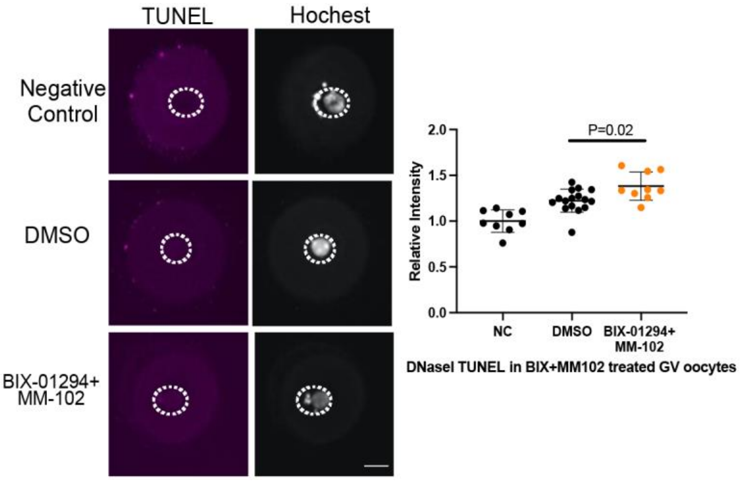

Figure DNaseI-TUNEL assay of oocytes were treated with BIX-01294 and MM-102.

Also, published research shows that chromatin accessibility can be altered by the inhibitor of G9a/GLP (Schones et al. 2014. Epigenetics & Chromatin). In addition to the new results, we have already mentioned other possibilities of changing chromatin accessibility (see discussion in **line 510-518**).
In summary, we have added new data to support our mechanistic model by showing that Zscan4 is clearly under the regulation of Histone marks and global chromatin status, and inhibitors do cause aberrant chromatin accessibility.

© 2022. Published by The Company of Biologists under the terms of the Creative Commons Attribution License (https://creativecommons.org/licenses/by/4.0/).

We have revised the text and added the new results in **new Fig. 6A and Fig. S6D**. We have also modified our figure of the model (**Fig 6G**) by adding chromatin status below the histone marks.

Lastly, 'Overexpression of Zscan4 in the oocytes recapitulates the phenotypes of Hira mutants' should be 'Overexpression of Zscan4 partially recapitulates the phenotypes of Hira mutants'.

We have corrected it accordingly.

3. line152
>Thus, the Hira complex is responsible for the acquisition of developmental competence in mouse oocytes, the corresponding genes functioning as maternal-effect genes.
I couldn't understand what does 'the corresponding genes functioning as maternal-effect genes' mean.
Better to remove.

Also, "responsible" is not appropriate, as Hira complex is one of many requirements for developmental competence. I recommend the term "required" instead of "responsible".

Sorry for the confusion, we have revised this.

4. line 222
>We chose to focus on Zscan4 as a candidate gene since it is involved in telomere maintenance.
"Candidate" for what means? Does this mean that Zscan4 is a candidate derepressed gene that might account for developmental failure?

We apologise for the lack of clarity. We have revised the text. See **line 195-198.**

5. Fig 3A/B.
Zscan4 is upregulated in ZH KO and CabZ KO oocytes. Since Zscan4 is known to be regulated by DUX, does the authors check if DUX is also upregulated? Regardless of the result, this information deserves to be included.

Many thanks for the Reviewer's suggestion.

We have checked the expression of Dux (Duxf3) and Dux family (Duxf1, Duxf2, Duxf4, and Dux5) in our ZH and CabZ GV oocyte RNA-seq datasets. Overall, the FPKM values are very low to undetectable in both ZH and CabZ Het/KO GV oocytes. One reported oocyte-expressed Duxf4=GM4981 (Chen and Zhang 2019) is indeed expressed.

In addition, we also adapted the RT-PCR approach using primers against Dux family reported by Sugie et al, (2020 Scientific Report) to examine Dux expression in the CabZ Het/KO, ZH Het/KO GV oocytes and Control/Ubn1 MO injected oocytes. We did not amplify Dux products in the oocytes.

Despite this we cannot formally exclude the possibility that the Dux family is involved in the regulation of Zscan4 in the oocyte. The possibility of Dux regulating Zscan4 seems unlikely given the very low-to-undetectable levels in the GV oocytes.

We have now added the values to **New Fig. S2C** and mentioned it in the main text (in **line 205-209**.)

	AW822073 (Duxf1)	Gm10807 (Duxf2)	Dux (Duxf3)	Gm4981 (Duxf4)	Gm19459 (Duxf5)
ZH Het	0.070	0.010	0.047	10.795	0.010
	0.010	0.206	0.010	14.043	0.010
	0.010	0.086	0.010	14.812	0.010
	0.010	0.134	0.010	10.650	0.057
ZH KO	0.010	0.010	0.081	5.405	0.132
	0.169	0.088	0.033	13.484	0.069
	0.010	0.200	0.038	4.481	0.153
	0.148	0.010	0.236	6.536	0.080

© 2022. Published by The Company of Biologists under the terms of the Creative Commons Attribution License (https://creativecommons.org/licenses/by/4.0/).

	AW822073 (Duxf1)	Gm10807 (Duxf2)	Dux (Duxf3)	Gm4981 (Duxf4)	Gm19459 (Duxf5)
CabZ Het	ND	ND	ND	0.425	ND
	ND	ND	ND	0.395	ND
	ND	ND	ND	0.346	ND
	ND	ND	ND	0.252	ND
CabZ KO	ND	ND	ND	0.352	ND
	ND	ND	ND	0.206	ND
	ND	ND	ND	0.283	ND
	ND	ND	ND	0.249	ND

NOTE: We have removed unpublished data that had been provided for the referees in confidence.

6. Fig 3C/D. Is the quantification data measuring the signals only in SN oocytes? If so, state it in figure legends. If not, quantification of only SN oocytes is necessary because SN and NSN are very different in transcriptional activity.

We apologise for not providing the details. To clarify this point, we have now revised and added an additional description into the figure legend, already written in the M&M Immunofluorescence section.

7. line 269
>Recent research indicates that two main histone repressive marks in this context, H3K9me3 and H3K4me3 (Dahl et al., 2016), (Zhang et al., 2016), are responsible for the transcriptional quiescence in SN oocytes (Dumdie et al., 2018a).
I am not aware that these papers demonstrate the responsibility of H3K9me3 in transcriptional quiescence in oocytes (H3K4me3 was demonstrated). So, need to rephase this part.

We apologise for this misunderstanding. We have revised it. Please see **line 260-261**.

8. Fig S6.
Data of the inhibitor-mediated derepression of Zscan4 is one of the main results in this story. So, I recommend to move the Fig S6A-red (mixed inhibitor experiment) to a main figure.

Additionally, since the bar graphs which lack bars of the control groups are not easy to understand, the bar of control groups should be added.

Thanks for the comment. We have added the bar to the control group, generated a new figure (changed the Y-axis to fold change) and moved the figure **(new Fig. 6A)**.

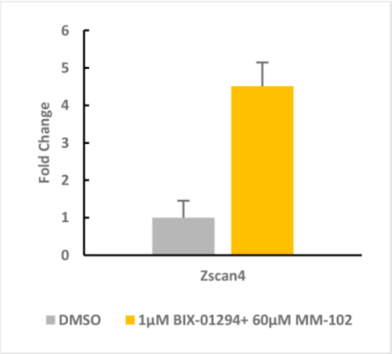

Figure qRT-PCR of Zscan4 after of oocytes treated with BIX-01294 and MM-102.

© 2022. Published by The Company of Biologists under the terms of the Creative Commons Attribution License (https://creativecommons.org/licenses/by/4.0/).

9. line 405
Fig 6B, not Fig 5B.

Thanks for the comment. We have corrected it.

10. line 425
Fig 6E, not 6C

Thanks for the comment. We have corrected it.

Second decision letter

MS ID#: DEVELOP/2021/200044

MS TITLE: The H3.3 chaperone Hira complex orchestrates oocyte developmental competence

AUTHORS: Rowena Smith, Andrej Susor, Hao Ming, Janet Tait, Marco Conti, Zongliang Jiang, and Chih-Jen Lin

I have now received all the referees' reports on the above manuscript, and have reached a decision. The referees' comments are appended below, or you can access them online: please go to BenchPress and click on the 'Manuscripts with Decisions' queue in the Author Area.

As you will see, the referee 2 still have some significant criticisms of the revised version and recommend further editorial and substantial revision of your manuscript before we can consider publication. If you are able to revise the manuscript along the lines suggested, which may involve further experiments, I will be happy receive a revised version of the manuscript. Your revised paper will be re-reviewed by one or more of the original referees, and acceptance of your manuscript will depend on your addressing satisfactorily the reviewers' major concerns. Please also note that Development will normally permit only one round of major revision.

We are aware that you may be experiencing disruption to the normal running of your lab that make experimental revisions challenging. If it would be helpful, we encourage you to contact us to discuss your revision in greater detail. Please send us a point-by-point response indicating where you are able to address concerns raised (either experimentally or by changes to the text) and where you will not be able to do so within the normal timeframe of a revision. We will then provide further guidance. Please also note that we are happy to extend revision timeframes as necessary.

Please attend to all of the reviewers' comments and ensure that you clearly highlight all changes made in the revised manuscript. Please avoid using 'Tracked changes' in Word files as these are lost in PDF conversion. I should be grateful if you would also provide a point-by-point response detailing how you have dealt with the points raised by the reviewers in the 'Response to Reviewers' box. If you do not agree with any of their criticisms or suggestions please explain clearly why this is so.

Reviewer 1

Advance summary and potential significance to field

In this manuscript, the authors investigate the role of maternal Hira complex in oocyte developmental competence. They found that knockout or knockdown of any subunit of the Hira complex during mouse oocyte development leads to early embryogenesis failure. Mechanistically, embryos without the Hira complex fail to silence global transcription, due to compromised H3.3 deposition, reduced repressive H3K4me3 and H3K9me3 histone marks, and aberrant chromatin accessibility. Moreover, the authors demonstrated that the elevated expression of Zscan4 in these mutant embryos is responsible for the embryogenesis failure. Overall, the role and mechanism of

© 2022. Published by The Company of Biologists under the terms of the Creative Commons Attribution License (https://creativecommons.org/licenses/by/4.0/).

the Hira complex in regulating oocyte developmental competence is of significance for the field of developmental biology.

Comments for the author

The authors have addressed my comments. Thus, I recommend to publish the paper.

Reviewer 2

Advance summary and potential significance to field

I see that the authors significantly revised the manuscript, which makes their conclusions more convincing than previously. Although I am generally supportive for this work to be publication in Development, I still feel that some statements are not appropriate and overpersuaded.

Comments for the author

Line 142-143
'Hira and Cabin1 pause expression of ~'. This sounds that Hira and Cabin1 directly pause transcription.
However, there is no data supporting it, but transcriptional derepression is very likely a downstream consequence of their depletion. So, this sentence should be more modest, like 'Several ZGA genes are derepressed in Hira and Cabin1 KO oocytes'.
line172
'To exclude the possibility that ~'.
I cannot understand why they need to exclude the possibility. 'To address the possibility that ~' is OK.
line176
'~ minimal overlapping with NSN oocytes'
Does this mean 'minimal overlapping with genes downregulated in SN oocytes'?
Line 244
"in both ZH and CabZ mutant"
This is not correct, because Gm5039 is not upregulated in Hira KO. "in ZH or CabZ mutant" is OK.
Line247-248 (Fig S3C)
This figure is difficult to understand. Which bars should be compared to which? Why some are 1PN zygotes?
What is the purpose of this bar graph? I don't think this graph add anything in this story. So, I recommend to remove it.
Line253-254
'Two key repressive histone marks, H3K4me3 and H3K9me3, are not ~'
This statement is confusing, as H3K4me3 is generally an active mark (even in oocytes, it actually marks active genes). Remove this and just say 'H3K4me3 and H3K9me3 are not ~'. Also, remove "two histone repressive marks" at line 258.
Line 258-259 Which paper shows that H3K9me3 is critical for developmental competence?
Line 372-374
'the up-regulation of Zscan4 was similar to ~'
I don't think this is "similar". For example, IF in fig 3C,D shows very mild upregulation of Zscan4 in KO oocytes, while those in S6C shows very clear enrichment in OE. I really feel this overpersuaded, so I strongly suggest removing this statement.
Line 480-481
'mutant oocytes lack H3K4me3/H3K9me3 marks'
They don't actually "lack" them. Rephrase to 'H3K4me3/H3K9me3 are decreased in Hira and Cabin1 mutant oocytes.'
Fig 6A The H3K9me3 IF signal is too faint to be seen. Increase the signal intensity.
Fig S7A The Zscan4 IF signal is too faint to be seen. Increase the signal intensity.

© 2022. Published by The Company of Biologists under the terms of the Creative Commons Attribution License (https://creativecommons.org/licenses/by/4.0/).

Second revision

Author response to reviewers' comments

Point-to-Point Response

We would like to thank both Reviewers again for their valuable comments (in **bold black colour**), which have helped us improve the manuscript significantly. We have addressed the points (in blue colour) raised by each of the Reviewers with new data, as described below.

Reviewer 1 Comments for the Author:
The authors have addressed my comments. Thus, I recommend to publish the paper.

We would like to thank Reviewer 1 for the recommendation.

Reviewer 2 Comments for the Author:
Line 142-143
'Hira and Cabin1 pause expression of ~'. This sounds that Hira and Cabin1 directly pause transcription. However, there is no data supporting it, but transcriptional derepression is very likely a downstream consequence of their depletion. So, this sentence should be more modest, like 'Several ZGA genes are derepressed in Hira and Cabin1 KO oocytes'.

Thanks for the comment. We have corrected it accordingly.

line172
'To exclude the possibility that ~'.
I cannot understand why they need to exclude the possibility. 'To address the possibility that ~' is OK.

Thanks for the comment. We have corrected it accordingly.

line176
'~ minimal overlapping with NSN oocytes'
Does this mean 'minimal overlapping with genes downregulated in SN oocytes'?

Thanks for the comment. We have revised to "NSN stage specifically expressed genes".

Line 244
"in both ZH and CabZ mutant"
This is not correct, because Gm5039 is not upregulated in Hira KO. "in ZH or CabZ mutant" is OK.

Thanks for the comment. We have corrected it accordingly.

Line247-248 (Fig S3C)
This figure is difficult to understand. Which bars should be compared to which? Why some are 1PN zygotes?
What is the purpose of this bar graph? I don't think this graph add anything in this story. So, I recommend to remove it.

Thanks for the comment. We have removed it accordingly.

Line253-254
'Two key repressive histone marks, H3K4me3 and H3K9me3, are not ~'
This statement is confusing, as H3K4me3 is generally an active mark (even in oocytes, it actually marks

© 2022. Published by The Company of Biologists under the terms of the Creative Commons Attribution License (https://creativecommons.org/licenses/by/4.0/).

active genes). Remove this and just say 'H3K4me3 and H3K9me3 are not ~'. Also, remove "two histone
repressive marks" at line 258.

Thanks for the comment. We have corrected it accordingly.

Line 258-259
Which paper shows that H3K9me3 is critical for developmental competence?

Thanks for the comment and we have cited a reference (Au Yeung et al., 2019) which reported that the H3K9me2/3 (by depletion of their methyltransferase, G9a) is essential for development.

Line 372-374
'the up-regulation of Zscan4 was similar to ~'
I don't think this is "similar". For example, IF in fig 3C,D shows very mild upregulation of Zscan4 in KO oocytes, while those in S6C shows very clear enrichment in OE. I really feel this overpersuaded, so I strongly suggest removing this statement.

Thanks for the comment. We have corrected it accordingly.

Line 480-481
'mutant oocytes lack H3K4me3/H3K9me3 marks'
They don't actually "lack" them. Rephrase to 'H3K4me3/H3K9me3 are decreased in Hira and Cabin1 mutant oocytes.'

Thanks for the comment. We have corrected it accordingly.

Fig 6A
The H3K9me3 IF signal is too faint to be seen. Increase the signal intensity.

Thanks for the comment. We have adjusted it accordingly.

Fig S7A
The Zscan4 IF signal is too faint to be seen. Increase the signal intensity.

Thanks for the comment. We have adjusted it accordingly.

<u>Third decision letter</u>

MS ID#: DEVELOP/2021/200044

MS TITLE: The H3.3 chaperone Hira complex orchestrates oocyte developmental competence

AUTHORS: Rowena Smith, Andrej Susor, Hao Ming, Janet Tait, Marco Conti, Zongliang Jiang, and Chih-Jen Lin
ARTICLE TYPE: Research Article

I am happy to tell you that your manuscript has been accepted for publication in Development, pending our standard ethics checks.

© 2022. Published by The Company of Biologists under the terms of the Creative Commons Attribution License (https://creativecommons.org/licenses/by/4.0/).

Reviewer 2

Advance summary and potential significance to field

NA

Comments for the author

The authors fully addressed my comments.

© 2022. Published by The Company of Biologists under the terms of the Creative Commons Attribution License (https://creativecommons.org/licenses/by/4.0/).
